# Immunomodulatory Nanozymes Eradicate Intracellular Infections and Rescue Immunoparalysis for Treating Multidrug‐Resistant Bacterial Sepsis

**DOI:** 10.1002/EXP.20250127

**Published:** 2025-06-01

**Authors:** Xuancheng Du, Zhenzhen Dong, Yan Yan, Yuan Gong, Meili Yuan, Chengtai Ma, Lingqi Xu, Yuanyuan Qu, Minhan Qu, Peng Pan, Weifeng Li, Wenyan Hao, Yingyi Yang, Xiangdong Liu, Mingwen Zhao, Zhenjiang Bai, Jiang Huai Wang, Jian Wang, Yong‐Qiang Li, Huiting Zhou

**Affiliations:** ^1^ Institute of Pediatric Research Children's Hospital of Soochow University Suzhou China; ^2^ Institute of Advanced Interdisciplinary Science School of Physics, Shandong University Jinan China; ^3^ Department of Critical Care Medicine Renmin Hospital of Wuhan University Wuhan China; ^4^ Department of Pediatric Surgery Affiliated Hospital of Zunyi Medical University Zunyi Guizhou China; ^5^ Department of Surgery UT Southwestern Medical Center Dallas Texas USA; ^6^ Department of Academic Surgery University College Cork Cork University Hospital Cork Ireland

**Keywords:** bacterial infections, immunosuppression, multidrug resistance, nanozymes, sepsis

## Abstract

Sepsis and their sequelae are the leading causes of death in intensive care units, with limited therapeutic options. Immunoparalysis plays a vital role in the pathophysiological progression of sepsis, leading to intracellular persistent infections and high mortality of septic patients. Eradicating intracellular infections and rescuing immunoparalysis are critical for sepsis management, yet effective tactics remain elusive. Here, we report immunomodulatory nanozymes (named PdIr@OMVs) that enable intracellular bacteria elimination and reinvigorate systemic innate‐adaptive immune response during immunoparalysis to tackle multidrug‐resistant (MDR) bacterial sepsis. The PdIr@OMVs are designed by encapsulating plasmonic PdIr nanocatalysts with immunostimulants of biocompatible bacterial outer membrane vesicles (OMVs). PdIr@OMVs exhibit unique localized surface plasmon response‐enhanced peroxidase‐like catalytic activity, and inherit the remarkable immunocyte‐targeting capability and adjuvanticity of OMVs. We demonstrate that PdIr@OMVs not only potentiate the phagolysosomal killing effect of impaired macrophages via in situ catalysis to eradicate intracellular MDR bacteria and burst antigen release, but also allow rapid activation/maturation of dendritic cells to boost the presentation of bacterial antigen and orchestrate innate‐adaptive immunity for rescuing immunoparalysis. In two immunocompromised mouse models of MDR bacterial sepsis, PdIr@OMVs collaboratively reduce bacterial burden and restore immune homeostasis, thereby circumventing organ damage and enabling the recovery of septic mice. Our work offers a promising therapeutic modality for sepsis and septic shock.

## Graphical abstract

Immunomodulatory nanozymes enable in situ macrophages engineering and orchestrate systemic innate‐adaptive immune response to eradicate intracellular bacteria and rescue immunoparalysis for treating multidrug‐resistant bacterial sepsis.

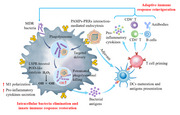

## 1 Introduction

Sepsis, a life‐threatening organ dysfunction syndrome caused by dysregulated host response to infection, affects more than 49 million people worldwide every year and results in nearly 20% of global deaths [1, 2, 3]. Recent clinical data reveal that more than 60% of septic patients survive through an initial inflammatory storm but rapidly progress to a longer systemic immunosuppressive state (immunoparalysis), leading to septic shock with intracellular persistent infections and high mortality [4, 5, 6]. Consequently, immunomodulatory therapies have been increasingly explored to tackle immunoparalysis and treat sepsis [7, 8]. Several small clinical trials of immunostimulatory agents (e.g. immunostimulatory cytokines and negative costimulatory molecule inhibitors) have shown the reversal of immunocyte dysfunctions and enhanced elimination of the invaded pathogens during sepsis‐induced immunoparalysis [9, 10]. However, meta‐analysis of large clinical trials have failed and show insignificant changes in reducing sepsis mortality [11]. Differences in clinical outcome may be explained as follows. First, conventional immunostimulatory agents are not able to restore paralyzed host immunity to normal levels [12]. Second, bacteria, the primary pathogens of sepsis, can evolve immune escape mechanisms during immunoparalysis, thereby permitting their intracellular survival and causing persistent infection, which is refractory and become devastating due to the prevalence of multidrug‐resistant (MDR) bacteria [13, 14, 15]. In this context, innovative tactics to eradicate intracellular MDR bacteria and rescue immunoparalysis are urgently needed for sepsis management.

Macrophages, essential components of the innate immune system, constitute the first line of defense against microbial invasion via phagocytosis and phagolysosomal killing, and form a bridge with the adaptive immune arm to regulate host immune homeostasis [16]. In septic patients, impaired macrophages with deficient antimicrobial function are primarily responsible for bacteria immune escape and intracellular infection, and play pivotal roles in the formation of immunoparalysis [6, 17], thus providing a valid target to tackle refractory sepsis. Several recombinant proteins and small‐molecule inhibitors have been demonstrated to potentiate the phagocytic function of macrophages in sepsis [18, 19]. Moreover, ingenious nanosystems have been developed to elevate the phagolysosomal killing capability of impaired macrophages via targeted delivery of antimicrobial peptide genes and bactericidal metal ions [20, 21, 22]. These agents significantly enhance the host innate immunity and enable efficient ablation of invaded bacteria for the treatment of bacterial sepsis; however, they are incapable of boosting systemic adaptive immune response and restoring immune homeostasis during immunoparalysis due to insufficient antigen presentation, thus significantly hindering their clinical application [21, 22]. Therapeutic strategies to collaboratively reinvigorate the antimicrobial activity of impaired macrophages and boost systemic innate‐adaptive immune response in sepsis‐induced immunoparalysis are thus in their fancy, but remain substantially challenging.

**FIGURE 1:**
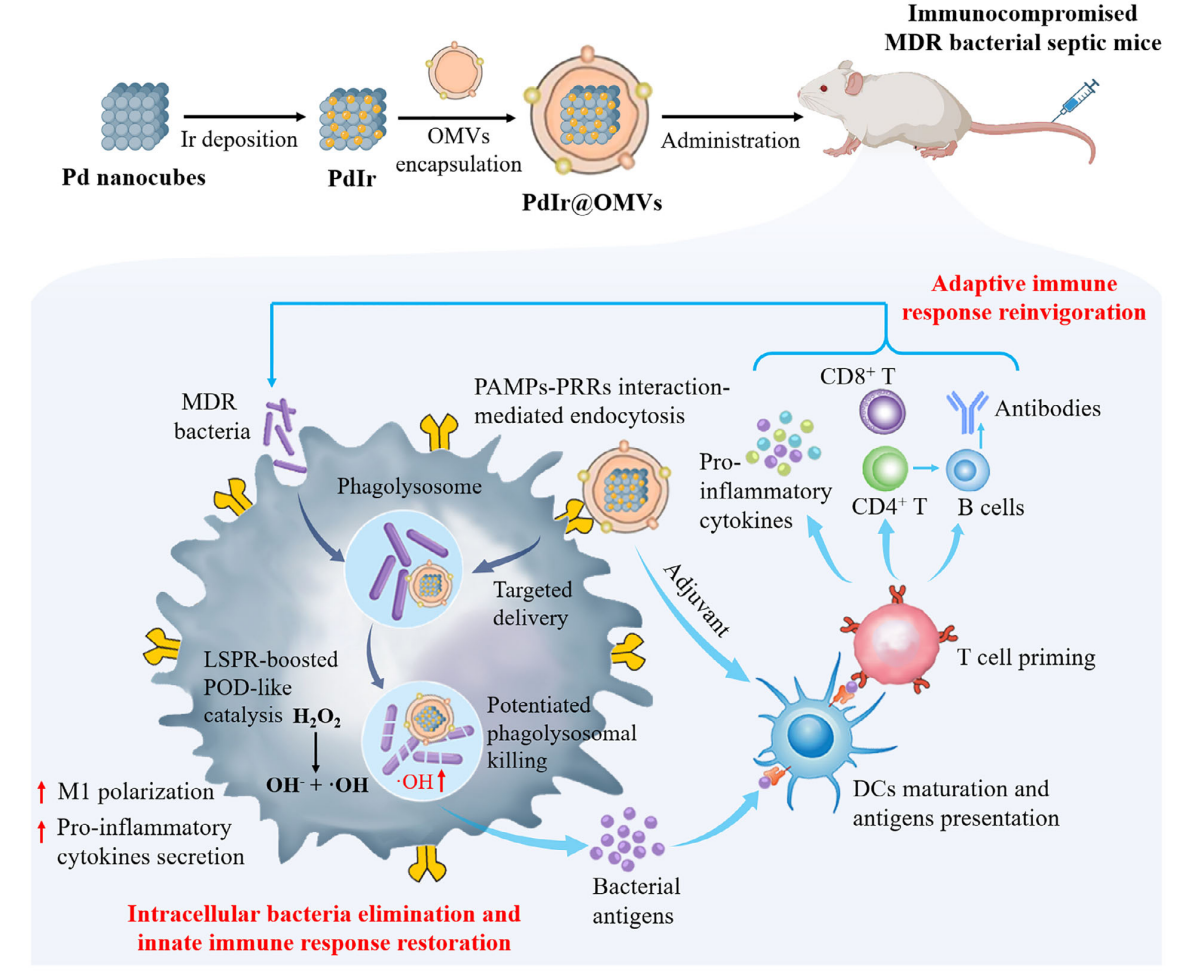
Schematic illustration of immunomodulatory nanozymes‐enabled in situ macrophages engineering and systemic innate‐adaptive immune response orchestration to eradicate intracellular bacteria and rescue immunoparalysis for MDR bacterial sepsis treatment.

Herein, we present immunomodulatory nanozymes (named PdIr@OMVs) that enable in situ macrophages engineering and orchestrate systemic innate‐adaptive immune response to eradicate intracellular bacteria and rescue immunoparalysis for MDR bacterial sepsis treatment (Figure 1). The immunomodulatory nanozymes (denoted as PdIr@OMVs) are constructed by coating de novo designed plasmonic PdIr nanocatalysts (PdIr) with bacterial outer membrane vesicles (OMVs). Nanozymes are a type of nanomaterials than function similarly to natural enzymes, and have receive extensive attention in sepsis treatment due to their robust antioxidant and antimicrobial effects [23, 24, 25]. By leveraging localized surface plasmon response (LSPR) [26], the PdIr nanocatalysts exhibit superior peroxidase (POD)‐like activity to catalyze hydrogen peroxide (H_2_O_2_) into bactericidal hydroxyl radical (·OH) and enable completely eradication towards MDR bacteria. OMVs secreted by gram‐negative bacteria have similar compositions as bacterial membrane and contain various immunostimulatory components acting as persistent pathogen‐associated molecular patterns (PAMPs) [27, 28, 29]. In MDR bacterial sepsis‐induced immunoparalysis, PdIr@OMVs can be recognized by impaired macrophages through PAMPs‐pattern recognition receptors (PRRs) interaction and are directionally transported into the phagolysosome to potentiate the phagolysosomal killing efficiency via in situ POD‐like catalytic reaction, thus bursting bacterial antigen release and reinvigorating innate immunity. Moreover, PdIr@OMVs inherit the immunostimulatory capability of OMVs and allow rapid activation/maturation of dendritic cells (DCs), thereby boosting the presentation of released bacterial antigen and T cell priming and evoking systemic adaptive immune response. Compared with reported nanozymes with only antioxidant and antimicrobial effects, PdIr@OMVs possessing LSPR‐enhanced POD‐like activity and robust immunomodulatory effect, simultaneously address the critical challenges of MDR bacterial infection and immunoparalysis, offering a promising paradigm for the treatment of sepsis and septic shock.

## 2 Results and Discussion

### 2.1 Preparation and Characterization of PdIr@OMVs

**FIGURE 2:**
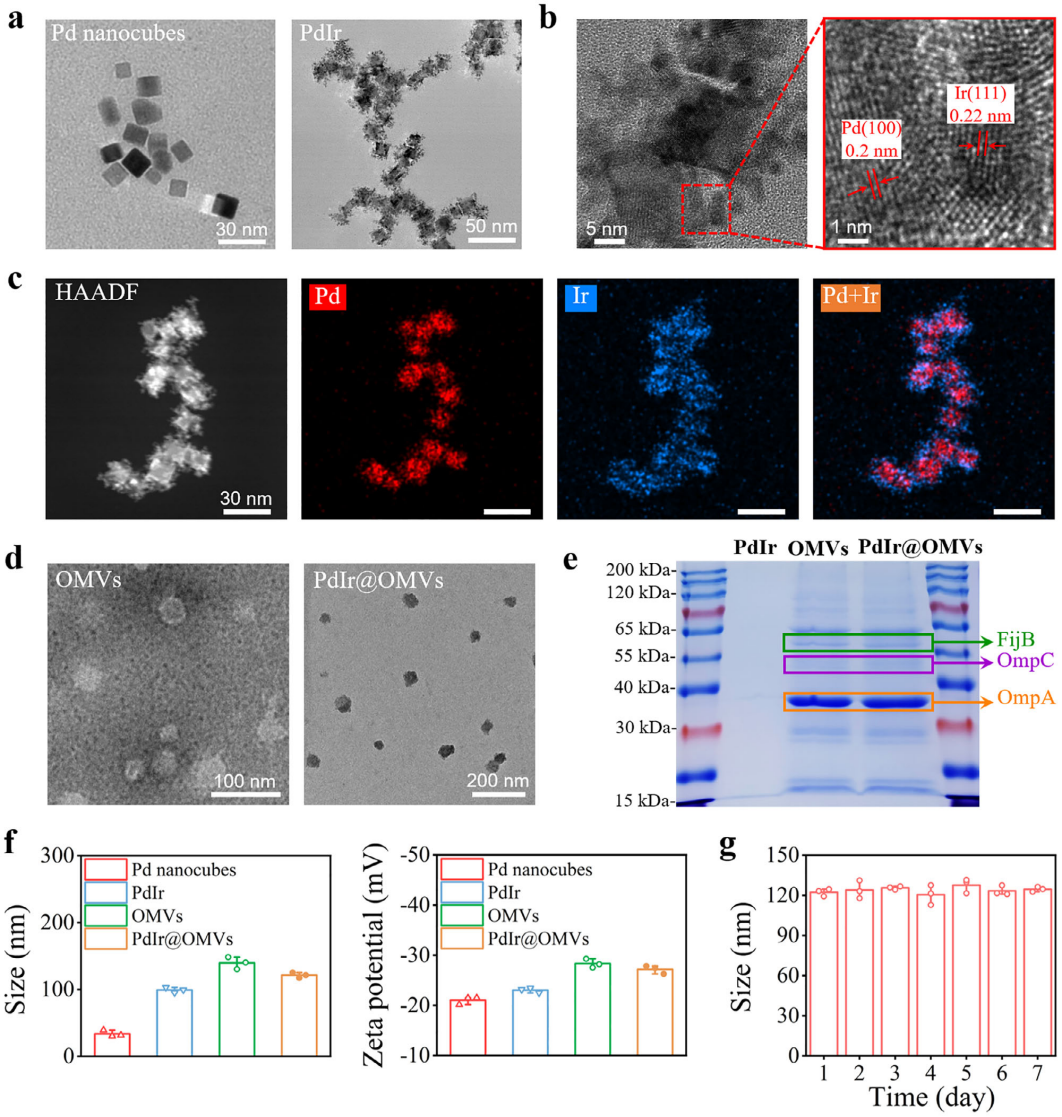
Characterization of PdIr@OMVs. (a) TEM images of Pd nanocubes, and PdIr. (b) High resolution TEM images of PdIr to show the crystal lattice. (c) EDX mapping of PdIr. (d) TEM images of OMVs and PdIr@OMVs. (e) SDS‐PAGE protein profiles of PdIr, OMVs, and PrIr@OMVs. (f) Hydrodynamic diameters and zeta potentials of Pd nanocubes, PdIr, OMVs, and PdIr@OMVs in DI water (*n* = 3). (g) Hydrodynamic diameter of MCeC@MΦ in DI water during 7 days of storage (*n* = 3).

The PdIr nanocatalysts were synthesized based on a facile seed‐mediated growth method by utilizing Pd nanocubes (size around 14 nm) as seeds and polyvinyl pyrrolidone (PVP) molecules as stabilizers [30, 31]. Transmission electron microscopy (TEM) images showed that Ir nanoparticles grew uniformly on the surface of well‐dispersed Pd nanocubes in PdIr nanocatalysts (Figure 2a). From the high‐resolution TEM images of PdIr nanocatalysts, the lattice spacings of Pd nanocubes and Ir nanoparticles were determined to be around 0.2 and 0.22 nm, respectively, corresponding to the Pd(100) and Ir(111) facets (Figure 2b) [30]. In addition, energy‐dispersive X‐ray (EDX) elemental mapping and X‐ray photoelectron spectrum (XPS) of PdIr all revealed the co‐existence of Pd and Ir elements, further verifying the successful preparation of PdIr nanocatalysts (Figure 2c and Figure S1, Supporting Information). Moreover, the doping ratios of Ir element in PdIr nanocatalysts were easily controlled by adjusting the molar mass of input Ir precursor. As shown in the TEM images of PdIr with different Ir doping ratios (Figure S2, Supporting Information), the deposition of Ir nanoparticles on Pd nanocubes increased continually with the initial Ir precursor concentration, which was consistent with the constantly elevated absorbance, shifted diffraction peaks, and increased hydrodynamic size results (Figures S3 and S4, Supporting Information).

The immunomodulatory nanozymes of PdIr@OMVs were then prepared by encapsulating synthesized PdIr nanocatalysts with OMVs through polycarbonate film extrusion [32, 33]. In our experiments, attenuated gram‐negative bacteria of *Escherichia coli* (*E. coli*)‐derived OMVs obtained by vacuum filtration and ultracentrifugation were employed [34]. As shown in Figure 2d and Figure S5, Supporting Information, the PdIr alloy cores were coated with thin shells, indicating the encapsulation of OMVs. Through OMVs coating, the PdIr@OMVs are expected to acquire the antigenic exterior of *E. coli*. To confirm this, sodium dodecyl sulfate‐polyacrylamide gel electrophoresis (SDS‐PAGE) was carried out to study the protein composition of PdIr@OMVs. As shown in Figure 2e, PdIr@OMVs presented a protein profile nearly exactly the same as that of OMVs, verifying the successful translocation of OMVs onto the PdIr nanocatalysts. In particular, the bands for the flagellin (FijB), outer membrane protein A (OmpA), and outer membrane protein C (OmpC), which represent the typical PAMPs existed in *E. coli* [35], could be recognized from the SDS‐PAGE protein profile. The expression of typical OMVs’ protein maker of OmpA was also clearly found in PdIr@OMVs from the Western blotting result, indicating the effective translocation of OMVs onto the PdIr nanocatalysts (Figure S6, Supporting Information). In addition, the loading concentrations of protein on PdIr@OMVs (100 µg of protein per 250 µg of PdIr) were calculated based on bicinchoninic acid (BCA) assay [36], which is basically the same as the purified OMVs, proving the successful synthesis of PdIr@OMVs (Figure S7, Supporting Information). Moreover, the whole preparation process of immunomodulatory nanozymes (from Pd nanocubes to PdIr@OMVs) could also be monitored and confirmed by the increased hydrodynamic size and zeta potential results (Figure 2f). Furthermore, the prepared PdIr@OMVs possessed outstanding hydrodynamic size stability (Figure 2g), which might be ascribed to the stabilizing effect of hydrophilic surface glycans on OMVs [32, 33].

### 2.2 Intrinsic POD‐Like Activity of PdIr@OMVs

**FIGURE 3:**
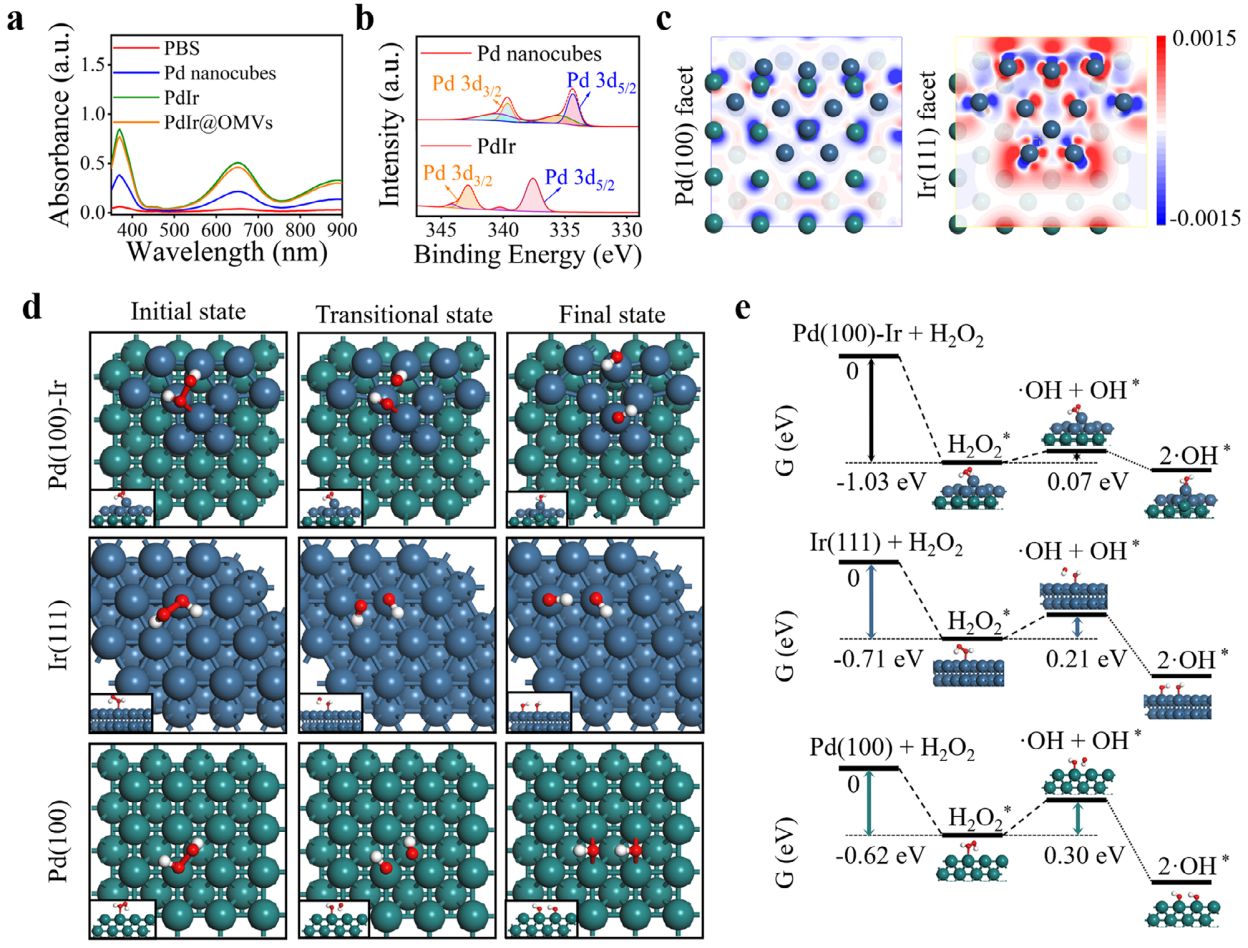
Intrinsic POD‐like activity of PdIr@OMVs. (a) Oxdation of TMB (reaction system: TMB + H_2_O_2_) in the group of Pd nanocubes, PdIr, and PdIr@OMVs. (b) Binding energy peaks of the Pd 3d_3/2_ and Pd 3d_5/2_ in PdIr. (c) Electron density difference maps of Pd(100) and Ir(111) facets in PdIr. (d) Schematic coordination configuration of all states (initial, transitional, and final state) during POD‐like catalytic processes on the surfaces of Pd(100), Ir(111), and Pd(100)‐Ir. (e) Gibbs free energy profiles of POD‐like reaction on the surfaces of Pd(100), Ir(111), and Pd(100)‐Ir. “*” represents the catalytic site. In (c), (d), and (e), green, blue, red, and white balls represent Pd, Ir, O, and H atoms, respectively.

As a typical platinum group metal, Pd nanocube has been reported to exhibit intrinsic POD‐like activity to catalyze the conversion of H_2_O_2_ into ·OH [37]. Since Pd and Ir have different Fermi levels, the incorporation of Ir into a confined Pd nanocube is theoretically effective to modulate the electronic structure of active center, which is expected to accelerate the electron transfer efficiency between Pd nanocube and substrate and boost its POD‐like activity. To demonstrate this, the POD‐like activity of PdIr nanacatalysts was systematically investigated based on 3,3',5,5'‐tetramethylbenzidine (TMB) oxidation assay. As the most representative substrate of POD reaction, colorless TMB could be oxidized into blue oxTMB in the presence of H_2_O_2_ [38, 39]. As shown in Figure 3a, significantly increased absorption of oxTMB was observed in group of PdIr compared to Pd nanocubes, indicating the boosted POD‐like activity of PdIr nanocatalysts. In addition, we examined the effect of Ir doping ratio on the POD‐like activity of PdIr nanocatalysts. As shown in Figure S8, Supporting Information, the POD‐like catalytic performance of PdIr nanocatalysts improved with the increasing doping ratio of Ir, and a suitable doping ratio of the molar ratio of Pd to Ir element at 1:1 was chosen in which nearly 4‐fold POD‐like activity than Pd nanocubes was obtained. Moreover, the POD‐like activity of PdIr nanocatalysts remained basically the same before and after OMVs coating, indicating that the encapsulation of OMVs did not affect the catalytic performance of PdIr possibly due to the permeability of OMVs (Figure 3a).

We subsequently investigated the mechanism of boosted POD‐like activity of PdIr nanocatalysts. From the XPS spectra of Pd nanocubes, Ir nanoparticles and PdIr nanocatalysts, it was found that the Pd 3d_3/2_ and Pd 3d_5/2_ peaks in PdIr were shifted to higher binding energy by 3.22 and 3.09 eV, respectively, compared to that in Pd nanocubes (Figure 3b and Figure S9, Supporting Information). This phenomenon indicated that the electrons in PdIr nanocatalysts might be transferred from Pd to Ir [40], and the accumulation of electrons on Ir atoms was beneficial for the POD‐like catalytic reaction. To further confirm this, the electron density difference maps of Pd(100) and Ir(111) facets in PdIr nanocatalysts were drawn based on density functional theory (DFT) calculation (Figure 3c). It was found that Pd atoms (green balls) exhibited lower electron density (blue color) on the Pd(100) facet, while higher electron density (red color) around Ir atoms (blue balls) was clearly observed on the Ir(111) facet. The significant difference in electron density between Pd and Ir atoms directly proves the electrons transfer from Pd to Ir in PdIr nanocatalysts [41]. Moreover, Gibbs free energy (G) of all states (initial, transitional, and final state) during POD‐like catalytic processes was calculated by DFT to explore the mechanism of boosted activity of PdIr nanocatalysts more comprehensively (Figure 3d,e). In our model, the Pd(100)‐Ir heterogeneous configuration was established as the optimal structure of PdIr by arranging Ir(111) atoms on the Pd(100) substrate (Figure 3d). As shown in Figure 3e, the adsorption energies of H_2_O_2_ on Ir(111), Pd(100) and Pd(100)‐Ir surfaces were calculated to be −0.71, −0.62, and −1.03 eV, respectively. This result demonstrated that H_2_O_2_ was more readily adsorbed on the Pd(100)‐Ir surface compared to the Ir(111) and Pd(100), which was favorable for the subsequent disassociation reaction. The energy barrier for the dissociation of H_2_O_2_ molecules on the Pd(100)‐Ir surface was then determined to be approximately 0.07 eV, which is much lower than that on the surfaces of Pd(100) and Ir(111), indicating that H_2_O_2_ can dissociate more conveniently into ·OH in PdIr nanocatalysts.

### 2.3 LSPR‐Boosted POD‐Like Activity of PdIr@OMVs

**FIGURE 4:**
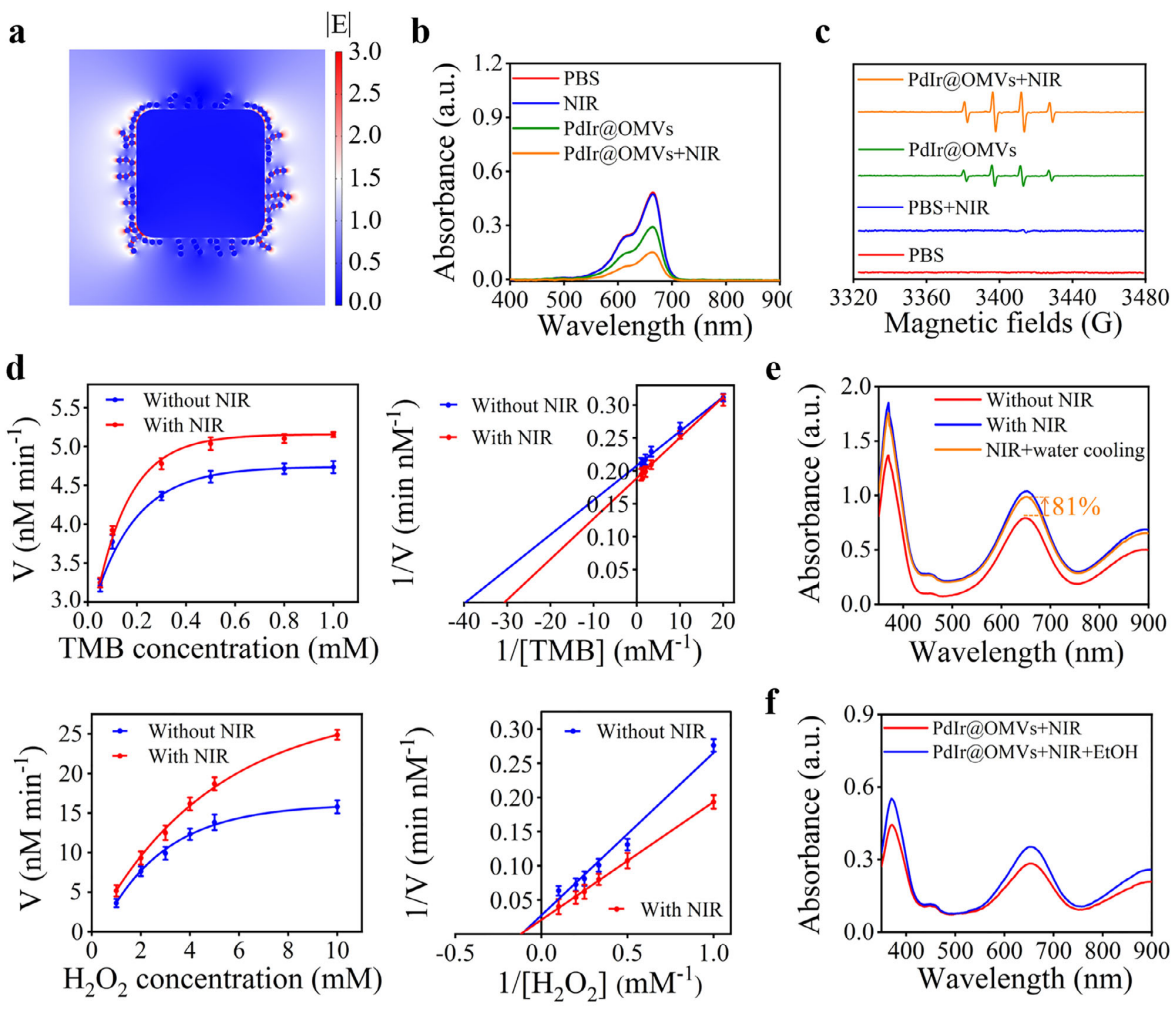
LSPR‐boosted POD‐like activity of PdIr@OMVs. (a) Theoretical simulation of the interaction between PdIr and incident electromagnetic wave upon NIR irradiation. (b) MB degradation (reaction system: MB + H_2_O_2_) in the presence of PdIr@OMVs with and without NIR irradiation. (c) ESR analysis (reaction system: DMPO + H_2_O_2_) in the presence of PdIr@OMVs with and without NIR irradiation. (d) Michaelis–Menten kinetics of POD‐like catalytic reaction of PdIr@OMVs with and without NIR irradiaiton using different substrates. The values of *V* and 1/*V* represent the mean of three independent experiments, and the error bars indicate the SD from the mean. (e) PdIr@OMVs‐catalyzed TMB oxidation in different experimental conditions. (f) PdIr@OMVs‐catalyzed TMB oxidation upon NIR irradiation in the absence/presence of hole scavenger of EtOH.

LSPR can be described as the collective oscillation of surface free electrons in plasmonic nanoparticles upon light irradiation, and it has been reported that hot electrons and local heating accompanied by the LSPR relaxation can improve the catalytic activity of nanozymes [26, 42, 43]. Pd and Ir nanoparticles have been considered as one of the most excellent plasmons, and the absorption spectra of PdIr exhibited strong absorption in the near‐infrared (NIR) region (Figure S3, Supporting Information). It is anticipated that LSPR would be excited on the surface of PdIr upon NIR irradiation, boosting their POD‐like catalytic activity. To verify this, theoretical simulation was first utilized to visualize the interaction between PdIr and incident electromagnetic wave upon NIR irradiation. As presented in Figure 4a, once a beam of laser with a wavelength of 808 nm was irradiated on plasmonic PdIr, the electric field was highly intensified and plenty of “hot spots” were formed on the surface of PdIr, indicating the occurrence of robust LSPR effect. To demonstrate the LSPR‐enhanced POD‐like activity of PdIr@OMVs, methylene blue (MB) degradation experiment was performed. During MB degradation, ·OH produced by POD‐like catalysis of PdIr@OMVs can attack the conjugated aromatic structure of MB and destroy its chromophore group, resulting in fading. As shown in Figure 4b, ·OH‐induced degradation of MB was greatly accelerated in the presence of PdIr@OMVs and H_2_O_2_ after NIR irradiation, implying that the POD‐like activity of PdIr@OMVs was boosted by LSPR to generate more ·OH for MB degradation. Electron spin resonance (ESR) analysis with typical quad signal peaks (intensity ratio: 1:2:2:1), directly confirmed the enhanced ·OH generation in the condition of PdIr@OMVs upon NIR irradiation (PdIr@OMVs + NIR) (Figure 4c) [44].

Subsequently, the steady‐state kinetics of POD‐like reaction of PdIr@OMVs were investigated. As shown in Figure 4d, PdIr@OMVs showed characteristic Michaelis–Menten kinetics of POD‐like catalytic reaction with and without NIR irradiaiton using H_2_O_2_ and TMB as substrate, respectively. The kinetic parameters (*V*_max_, *K*_M_, and *K*_cat_) were acquired from Lineweaver−Burk double reciprocal plots [45], and quantitative data on these parameters were listed in Table S1, Supporting Information. The results indicated that PdIr@OMVs upon NIR irradiation possessed much faster reaction velocity (1.1 and 1.34 times faster towards the substrate of TMB and H_2_O_2_, respectively) than that without NIR irradiation, directly proving the LSPR‐boosted POD‐like activity of PdIr@OMVs (Figure 4d and Table S1, Supporting Information). Hot electrons and photothermal effect accompanied by the relaxation of LSPR are both considered as the underlying mechanisms responsible for LSPR‐enhanced catalytic reaction [43]. To elucidate the proportional contribution of hot electrons and photothermal effect to the LSPR‐boosted POD‐like activity of PdIr@OMVs, TMB oxidation assay was carried out in which water cooling was employed to exclude the influence of local heating. As shown in Figure 4e, hot electrons were found to be the main contributor (81%) for the enhancement of POD‐like activity upon NIR irradiation. The low contribution rate of photothermal effect (19%) to the enhancement of POD‐like activity was mainly related to the relatively weak photothermal conversion capability of PdIr@OMVs (Figure S10, Supporting Information). In addition, by using ethanol (EtOH) as hole scavenger to decrease the recombination rate of photogenerated hot carriers [46], the POD‐like activity of PdIr@OMVs was significantly enhanced upon NIR irradiation, further proving the key role of hot electrons in LSPR‐boosted catalytic reaction of PdIr@OMVs (Figure 4f and Figure S11, Supporting Information). Moreover, the influence of pH and temperature on the POD‐like activity of PdIr@OMVs was comprehensively investigated. To our satisfaction, more than 80% of the POD‐like activity of PdIr@OMVs was retained at pH 5.5 and 37°C, which was beneficial for their application in cellular phagolysosomes, laying a solid foundation for subsequent phagolysosomal bacterial killing (Figure S12, Supporting Information).

### 2.4 In Vitro Bactericidal Performance of PdIr@OMVs

By leveraging the LSPR‐boosted POD‐like activity to catalyze H_2_O_2_ into bactericidal ·OH, PdIr@OMVs are expected to be a class of efficient bactericides. To confirm this, in vitro bactericidal performance of PdIr@OMVs was investigated. As shown in Figure S13, Supporting Information, PdIr@OMVs inhibited the growth of MDR *E. coli* by 70% in the presence of H_2_O_2_, and further completely stagnated bacterial growth upon NIR irradiation (PdIr@OMVs + H_2_O_2_ + NIR), possessing outstanding antimicrobial capability. To investigate the possible antibacterial mechanism of PdIr@OMVs, live/dead bacterial staining and scanning electron microscopy (SEM)‐based bacterial morphology study were carried out. As shown in Figure S14a,b, Supporting Information, MDR *E. coli* in the treatment groups of PdIr@OMVs + H_2_O_2_ + NIR, displayed obvious cellular deformations and surface collapse and were stained with propidium iodide (PI) dye (red fluorescence, which can only penetrate microorganisms with damaged structures) compared to the control (NIR, and H_2_O_2_). This phenomenon demonstrated the specific antimicrobial mechanism of PdIr@OMVs involving cell wall and membrane disruption [47]. Consistent with the results of live/dead staining and SEM, reactive oxygen species (ROS) burst (green fluorescence) in MDR *E. coli* was clearly observed in the treatment group of PdIr@OMVs + H_2_O_2_ + NIR, demonstrating that ROS burst caused by POD‐like catalysis of FdIr@OMVs would result in severe structure damage of bacteria and consequent killing (Figure S14c, Supporting Information). Biofilm is the predominant form of bacteria in vivo, and the eradication capability of PdIr@OMVs toward biofilm was also evaluated based on crystal violet staining [48]. As shown in Figures S15 and S16, Supporting Information, PdIr@OMVs not only inhibited the formation of biofilm, but also destroyed the mature biofilm of MDR *E. coli* in the presence of H_2_O_2_ under NIR irradiation, showing robust biofilm eradication capability.

### 2.5 PdIr@OMVs Potentiate the Phagolysosomal Killing Capability of Impaired Macrophages In Vitro

**FIGURE 5:**
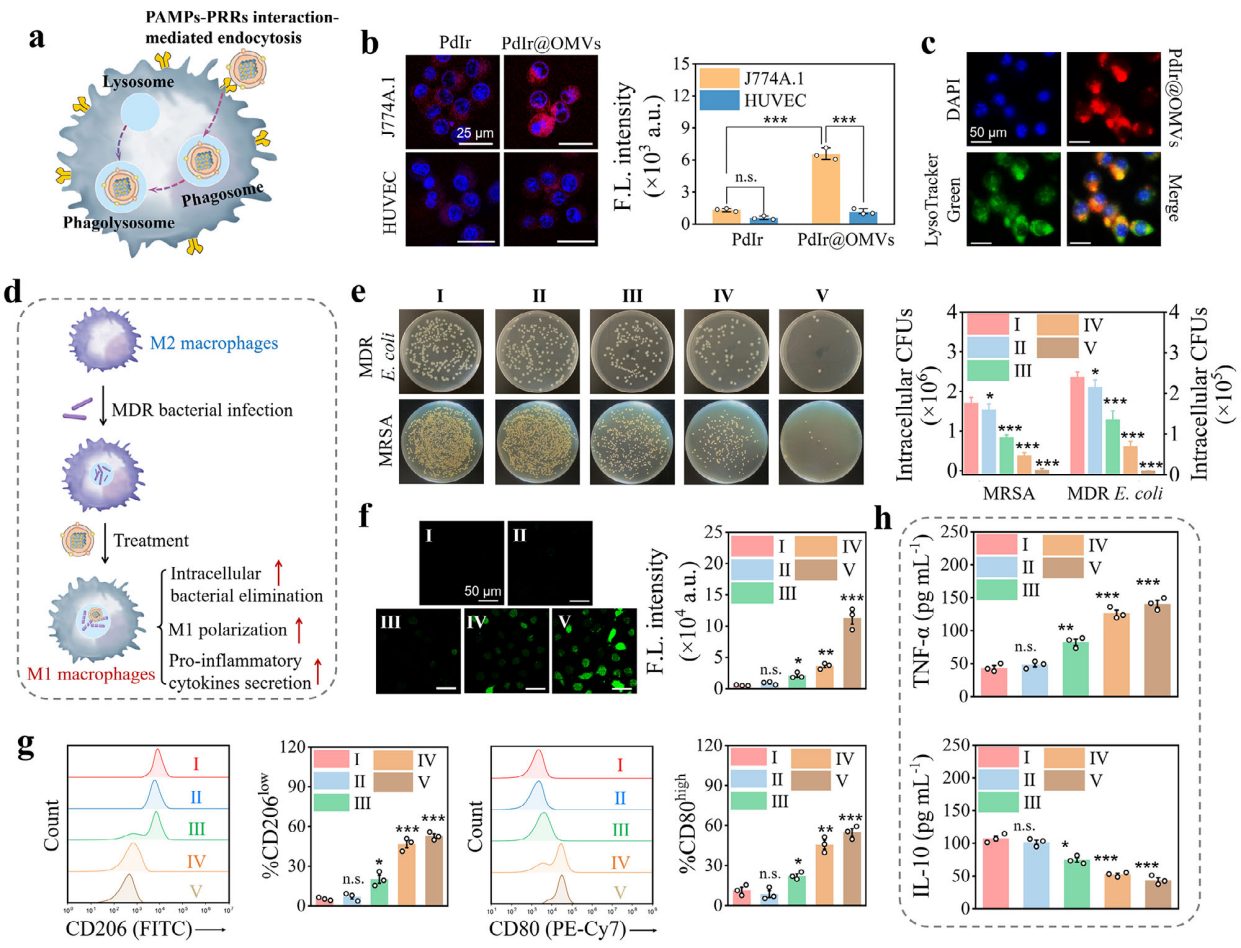
PdIr@OMVs potentiate the phagolysosomal killing of impaired macrophages to eliminate intracellular MDR bacteria and restore innate immune response in vitro. (a) Schematic illustration of the PAMPs‐PRRs interaction‐mediated macrophage recognition and targeted phagolysosome delivery of PdIr@OMVs. (b) Representative overlapping confocal fluorescence images and the corresponding fluorescence intensity of J774A.1 macrophages and HUVEC cells incubated with Cy5.5‐labeled PdIr and Cy5.5‐labeled PdIr@OMVs, respectively (*n* = 3). (c) Co‐localization assay of PdIr@OMVs and the lysosome in J774A.1 macrophages incubated with Cy5.5‐labeled PdIr@OMVs. (d) Schematic illustration of PdIr@OMVs‐potentiated phagolysosomal killing of infected M2 macrophage and innate immune response restoration. (e) Agar plate images of bacterial culture extracted from MDR *E. coli* and MRSA‐infected M2 J774A.1 macrophages upon different treatments, as well as the corresponding quantitative data of bacterial colonies (*n* = 3). (f) Intracellular ROS level of MDR *E. coli*‐infected M2 J774A.1 macrophages in different treatment groups (*n* = 3). (g) The expression of CD206 and CD80 by MDR *E. coli*‐infected M2 J774A.1 macrophages in different treatment groups (*n* = 3). (h) The secretion of TNF‐α and IL‐10 by MDR *E. coli*‐infected M2 J774A.1 macrophages in different treatment groups (*n* = 3). In (e), (f), (g), and (h), five treatment groups were employed including PBS (I), NIR (II), PdIr (III), PdIr@OMVs (IV), and PdIr@OMVs + NIR (V).

The demonstrated expression of PAMPs (i.e. FijB, OmpA, and OmpC) in PdIr@OMVs could empower them to be efficiently recognized by macrophages through PAMPs‐PRRs interaction and transported into the phagolysosome to initiate the phagolysosomal killing (Figure 5a). To test this, fluorescent PdIr@OMVs in which PdIr was labeled with Cy5.5 dye, were incubated with J774A.1 macrophages and human umbilical endothelial cells (HUVEC), respectively, and their cellular uptake was analyzed. As shown in Figure 5b and Figure S17, Supporting Information, J774A.1 macrophages incubated with fluorescent PdIr@OMVs showed much higher fluorescence than that incubated with PdIr, indicating the advantage of OMVs coating in enhanced macrophage‐associated recognition and cellular uptake. In sharp contrast, no fluorescence was found in HUVEC incubated with PdIr or PdIr@OMVs, pointing out the key role of PAMPs‐PRRs interactions in the recognition and cellular uptake of PdIr@OMVs by macrophages. The unique PAMPs‐PRRs interactions could further ensure the targeted delivery of PdIr@OMVs into the phagolysosome of macrophages. To clarify this, co‐localization assay of PdIr@OMVs and the lysosome in macrophages was performed. As shown in Figure 5c, nearly all PdIr@OMVs (red fluorescence) were co‐localized with the lysosome tracker (green fluorescence) in J774A.1 macrophages, confirming the specific accumulation of PdIr@OMVs in macrophage phagolysosomes.

The specific accumulation in macrophage phagolysosomes and superior POD‐like catalysis activity of PdIr@OMVs make them an ideal candidate for potentiating the phagolysosomal killing capability of impaired macrophages to eliminate intracellular bacteria and restore innate immune response. To demonstrate this, MDR bacteria‐infected M2 macrophages were constructed to mimic the impaired macrophages in sepsis‐induced immunoparalysis [49], and the impact of PdIr@OMVs treatment on elimination of intracellular bacteria, M1 macrophage polarization, and proinflammatory cytokines secretion was systematically evaluated (Figure S18, Supporting Information and Figure 5d). Figure 5e shows the agar plates of bacterial culture extracted from MDR *E. coli*‐ and methicillin‐resistant *Staphylococcus aureus* (MRSA)‐infected M2 J774A.1 macrophages upon different treatments as well as the corresponding quantitative data of bacterial colonies. It was found that intracellular MDR bacteria (*E. coli* and MRSA) in impaired macrophages were almost eradicated by PdIr@OMVs plus NIR irradiation (group V: PdIr@OMVs + NIR), indicating highly potentiated phagolysosomal killing capability. Consistent with the bacterial culture results, intracellular level of ROS especially •OH of infected M2 macrophages was substantially elevated in the PdIr@OMVs + NIR group, suggesting that the potentiated phagolysosomal killing capability of impaired macrophages could be attributed to in situ POD‐like catalysis of PdIr@OMVs (Figure 5f and Figure S19, Supporting Information). Of note, PdIr@OMVs greatly increased the intracellular level of H_2_O_2_ in infected M2 J774A.1 macrophages, which in turn promoted the efficiency of POD‐like catalysis of PdIr@OMVs (Figure S20, Supporting Information). The increased intracellular level of H_2_O_2_ was closely related to the recovery of immune response of impaired macrophages after PdIr@OMVs treatment. As shown in Figure S21, Supporting Information, the expression levels of key proteins of downstream NF‐κB pathway (p65, adaptor protein MyD88) and MAPK pathway (ERK, p38, JNK phosphorylation) in macrophages treated with PdIr@OMVs were significantly up‐regulated. This result indicated that by promoting the phagolysosomal killing for eliminating intracellular bacteria, PdIr@OMVs further activated downstream proinflammatory signaling pathways (NF‐κB and MAPK) to restore immune response in impaired macrophages. Be consistent with this, flow cytometry assay revealed that treatment with PdIr@OMVs plus NIR irradiation significantly upregulated the expression of CD80, a M1 macrophage marker, and down‐regulated the expression of CD206, a M2 macrophage marker in infected M2 J774A.1 macrophages, showing a positive effect on the polarization of impaired macrophages from M2 to M1 (Figure 5g). Moreover, the treatment of PdIr@OMVs plus NIR irradiation strongly enhanced the secretion of proinflammatory cytokine of tumor necrosis factor α (TNF‐α), and inhibited the secretion of anti‐inflammatory cytokine of interleukin‐10 (IL‐10) from infected M2 J774A.1 macrophages (Figure 5h and Figure S22, Supporting Information). Collectively, by promoting the phagolysosomal killing for eliminating intracellular bacteria, M1 macrophages polarization, and proinflammatory cytokine secretion, PdIr@OMVs enable the restoration of innate immune response in impaired macrophages.

### 2.6 PdIr@OMVs Promote the Maturation of DCs and Bridge the Innate and Adaptive Immune Response In Vitro

**FIGURE 6:**
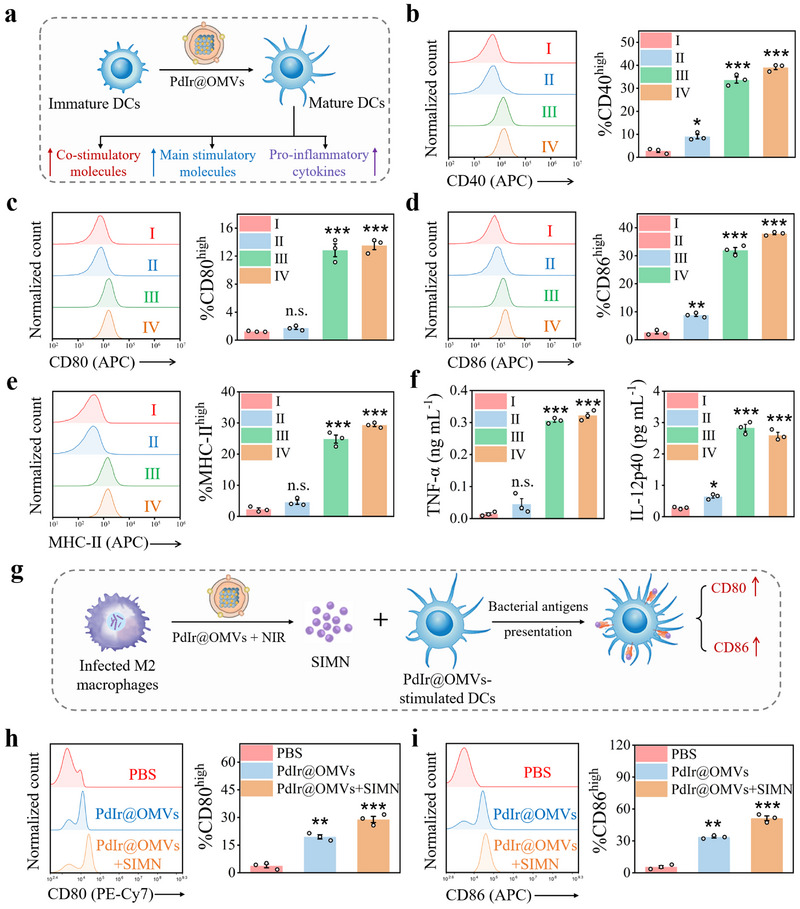
In vitro performance of PdIr@OMVs for promoting the maturation of DCs and bridging innate and adaptive immune response. (a) Schematic illustration of PdIr@OMVs‐promoted DCs activation and maturation. (b–e) The expression of CD40 (b), CD80 (c), CD86 (d), and MHC‐II (e) on DC2.4 cells on the 24th h of different treatments (*n* = 3). (f) The secretion of TNF‐α, and IL‐12p40 by DC2.4 cells on the 24th h of different treatments (*n* = 3). (g) Schematic illustration of PdIr@OMVs‐stimulated DCs for effective bacterial antigens presentation to bridge innate and adaptive immune response in vitro. (h, i) The expression of CD80 (h) and CD86 (i) on PdIr@OMVs‐stimulated DC2.4 cells with and without incubation with SIMN for 24 h (*n* = 3). In (b), (c), (d), (e) and (f), four treatment groups were employed including PBS (I), PdIr (II), OMVs (III), and PdIr@OMVs (IV).

The maturation of dendritic cells (DCs), a critical step for antigen presentation and T cell priming [50], is extremely suppressed in sepsis‐induced immunoparalysis, leading to paralyzed cooperation of innate and adaptive immunity. Due to the immunogenic antigens (PAMPs) present on the surface of OMVs, PdIr@OMVs are presumed to act as remarkable immune adjuvants for promoting the activation and maturation of DCs. To prove this, immature DCs was stimulated by PdIr@OMVs, and the effect of PdIr@OMVs on the activation and maturation of DCs was analyzed by evaluating the expression of co‐stimulatory molecules and main stimulatory molecules as well as the secretion of proinflammatory cytokines (Figure 6a). Flow cytometry assay revealed that PdIr@OMVs increased the expression of the co‐stimulatory molecules of CD40, CD80, and CD86 on DC2.4 cells by around 16, 9, and 19 times, respectively, compared to the control (PBS), showing remarkable adjuvanticity (Figure 6b–d). In addition, expression of main stimulatory molecule of major histocompatibility complex class II (MHC‐II) on DC2.4 cells stimulated by PdIr@OMVs was much higher (around 14 times) than that stimulated by PBS, suggesting promoted antigen presentation (Figure 6e). Furthermore, the secretion of proinflammatory cytokines of TNF‐α, interleukin‐6 (IL‐6), interleukin‐12p40 (IL‐12p40) and interkeukin‐1β (IL‐1β) by DC2.4 cells upon PdIr@OMVs stimulation was increased by 32, 25, 10, 2 times, respectively, in comparison with PBS (Figure 6f; Figures S23 and S24, Supporting Information). Of note, the expression of co‐stimulatory molecules/main stimulatory molecules and the secretion of proinflammatory cytokines upon OMVs stimulation, were identical to those upon PdIr@OMVs stimulation, confirming that the superior immunostimulatory capability of PdIr@OMVs is inherited from the encapsulation of OMVs (Figure 6b–f). The upregulated expression of co‐stimulatory molecules/ main stimulatory molecules and increased secretion of proinflammatory cytokines collectively confirmed the immunostimulatory performance of PdIr@OMVs for promoting the activation and maturation of DCs. The activation and maturation of DCs can further allow the effective presentation of bacterial antigens released from macrophages with potentiated phagolysosomal killing capability to evoke antigen‐specific adaptive immune response, thereby bridging innate and adaptive immunity. To test this, PdIr@OMVs‐stimulated DCs were incubated with the supernatant from MDR *E. coli*‐infected M2 macrophages treated by PdIr@OMVs plus NIR irradiation (SIMN) to assess the expression of the co‐stimulatory molecules (Figure 6g). As shown in Figure 6h,i, compared to PdIr@OMVs‐stimulated DC2.4 cells, the expression of the co‐stimulatory molecules of CD80, and CD86 on PdIr@OMVs‐stimulated DC2.4 cells after incubation with SIMN was increased by 1.5 and 1.6 times, respectively. These results indicate that bacterial antigens released from the infected macrophages treated by PdIr@OMVs can be effectively presented by PdIr@OMVs‐stimulated DCs, paving the way toward subsequent T cell priming and inducing orchestrated cooperation of innate and adaptive immune response.

### 2.7 Biocompatibility Assessment of PdIr@OMVs

The potential biotoxicity is a key obstacle restricting the practical applications of nanomaterials [51, 52], thus we evaluated the biocompatibility of PdIr@OMVs before performing in vivo experiments. As shown in Figure S25, Supporting Information, the viability of J774A.1, DC2.4 and HUVEC remained nearly 90% after incubation with PdIr@OMVs, suggesting that PdIr@OMVs would not induce apparent toxicity to both immune cells and normal cells in vitro. In addition, the hemolysis rate of PdIr@OMVs toward red blood cells (RBC) at around 0.2% was far lower than the international standard of 5%, proving the feasibility of intravenous (i.v.) injection (Figure S26, Supporting Information). Furthermore, healthy mice were intravenously administrated with PdIr@OMVs, and the tissue distribution of Pd and Ir elements, blood biochemical assay, and organ histopathological analysis were carried out on the 7th day of post‐injection to assess the biosafety of PdIr@OMVs in vivo. As shown in Figure S27, Supporting Information, a relatively higher amount of Pd and Ir elements was detected in the spleen and liver, indicating possible spleen and liver‐based metabolic pathway of PdIr@OMVs. There was no obvious difference in blood biochemical and routine indicators detected between the mice injected with PdIr@OMVs and control (PBS), indicating that PdIr@OMVs do not cause systemic inflammation and have negligible effect on the metabolism of liver and kidney of mice (Figures S28 and S29, Supporting Information). Additionally, histopathological analysis revealed that no inflammation and lesions were clearly observed in the organs of the mice administrated with PdIr@OMVs, exhibiting outstanding in vivo biocompatibility (Figure S30, Supporting Information). The demonstrated in vitro and in vivo biocompatibility of PdIr@OMVs lays a solid foundation for their subsequent MDR bacterial sepsis treatment.

### 2.8 PdIr@OMVs Rescue MDR *E. Coli*‐Induced Septic Mice With Immunoparalysis

**FIGURE 7:**
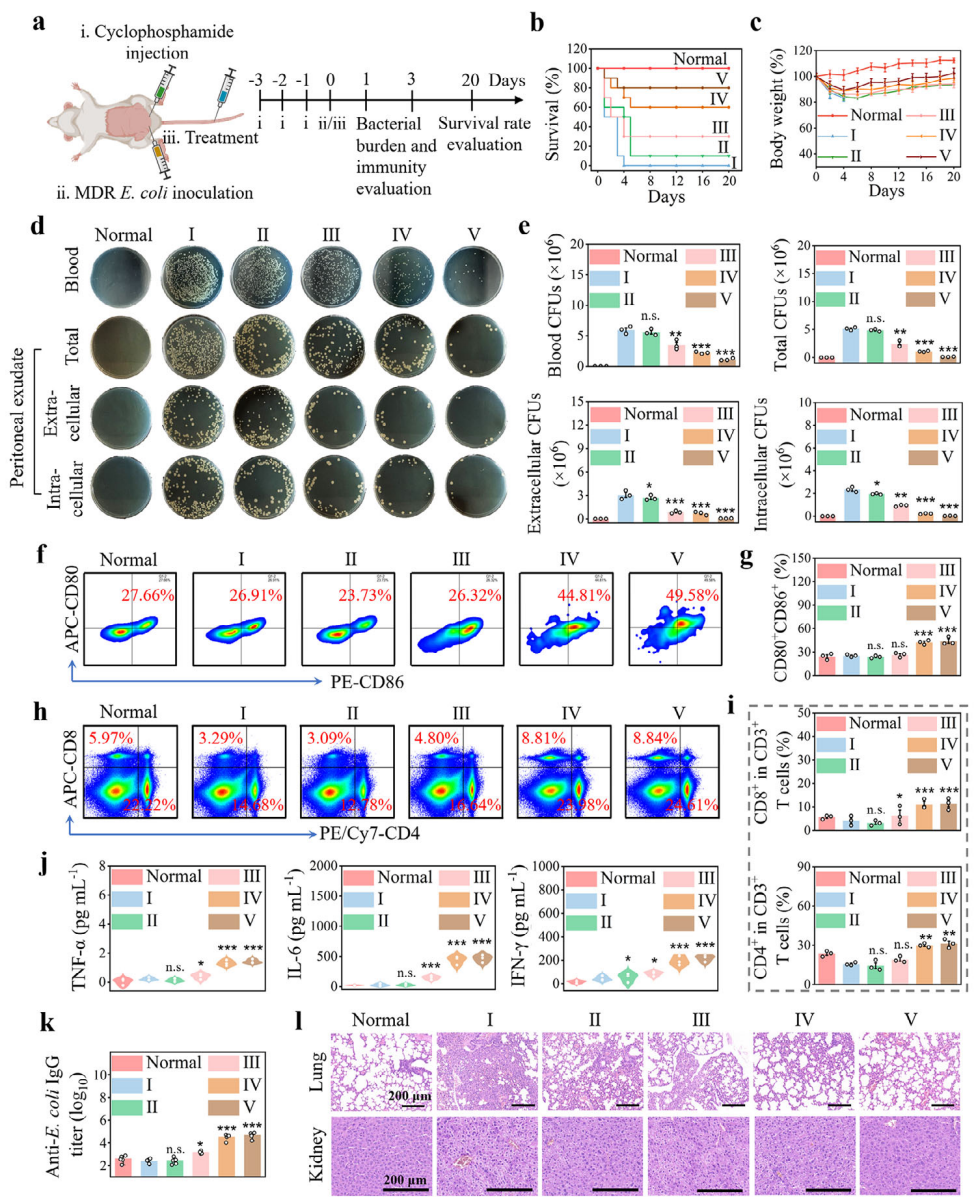
PdIr@OMVs rescue MDR E. coli‐induced septic mice with immunoparalysis. (a) Experimental timeline for the treatment of immunocompromised septic mice infected by MDR *E. coli*. (b, c) Survival rate (b) and body weight (c) of immunocompromised septic mice during 20 days of different treatments (*n* = 10). (d, e) Agar plate images of bacterial culture (d) extracted from the blood and peritoneal exudate of immunocompromised septic mice on the 24th h of different treatments, and the corresponding quantitative data of bacterial colonies (e). (f, g) Representative flow cytometric plots (f) and relative quantitative results (g) of matured DCs retrieved from the inguinal lymph nodes of immunocompromised septic mice on the 24th h of different treatments (*n* = 3). (h, i) Representative flow cytometric plots (h) and relative quantitative results (i) of cytotoxic T cells (CD3^+^CD8^+^) and helper T cells (CD3^+^CD4^+^) retrieved from the spleen of immunocompromised septic mice on the 24th h of different treatments (*n* = 3). (j) The secretion of TNF‐α, IL‐6, and IFN‐γ retrieved from the serum of immunocompromised septic mice on the 24th h of different treatments (*n* = 3). (k) Anti‐MDR *E. coli* lgG titer retrieved from the serum of immunocompromised septic mice on the 3rd day of different treatments (*n* = 3). (l) H&E staining images of the lung and kidney of immunocompromised septic mice on the 3rd day of different treatments. Treatment groups include PBS (I), NIR (II), PdIr (III), PdIr@OMVs (IV), and PdIr@OMVs + NIR (V), and healthy mice was used as the negative control.

The encouraging capabilities displayed by PdIr@OMVs in eradicating intracellular bacteria and reinvigorating innate‐adaptive immune response during immunosuppression make them ideal candidates for in vivo treatment of MDR bacterial sepsis. To evaluate the therapeutic performance of PdIr@OMVs, an immunoparalysis mouse model of MDR bacterial sepsis was first established by intraperitoneally administrating cyclophosphamide for three consecutive days and inoculating lethal doses of MDR *E. coli* (Figure 7a). Continuous i.p. administration of cyclophosphamide could effectively suppress the immune response of mice to result in immunoparalysis [53], as reflected by the significantly decreased white blood cells (WBCs) and loss in body weight (Figure S31, Supporting Information). Immunocompromised septic mice were then treated with different regimes 1 h after bacterial inoculation. Six groups (10 mice per group) were randomized divided: one control group consisted of healthy mice and six treatment groups including PBS, NIR, PdIr, PdIr + NIR, PdIr@OMVs, and PdIr@OMVs + NIR. As shown in Figure 7b and Figure S32a, Supporting Information, compared with a 100% mortality rate in PBS‐treated group, substantially improved survival was observed in the other four treatment groups with a maximal enhanced survival rate at 80% seen in PdIr@OMVs + NIR‐treated group. In addition, the body weight of immunocompromised septic mice treated with PdIr@OMVs + NIR began to recover on the 4th day post‐treatment and were closer to that of normal mice after 20 days of treatment, indicating the outstanding performance of PdIr@OMVs for in vivo intervention and management of MDR bacterial sepsis with immunoparalysis (Figure 7c and Figure S32b, Supporting Information).

To ascertain the mechanism underlying the therapeutic efficiency of PdIr@OMVs, bacterial burden of immunocompromised septic mice was first quantitatively assessed on the 24th h of treatment. It was found that the number of bacterial colonies in the blood of immunocompromised septic mice upon different treatments was positively correlated with the survival results, and bacteria were remarkably eliminated after the treatment of PdIr@OMVs + NIR (Figure 7d,e, and Figure S32c, Supporting Information). In addition to assessing the bacterial burden in the blood, bacterial numbers in the peritoneal exudate of immunocompromised septic mice upon different treatments were further determined. Since the peritoneal exudate of septic mice contains a large number of macrophages [54], both intracellular and extracellular bacterial burdens in the peritoneal exudate were analyzed, respectively, to evaluate the antimicrobial performance of PdIr@OMVs for intracellular bacteria. Notably, besides extracellular bacteria, intracellular bacteria in the peritoneal exudate of immunocompromised septic mice were found to be almost completely cleared in the treatment group of PdIr@OMVs + NIR, which is mainly attributed to the potentiated phagolysosomal killing by impaired macrophages, indicating the recovery of paralyzed innate immunity upon treatment (Figure 7d,e). Furthermore, we investigated the effect of PdIr@OMVs on neutrophils. Specifically, we extracted bone marrow neutrophils from immunosuppressive septic mice and examined the expression levels of their surface receptors NLRP1 and NLRP3. As shown in Figure S33, Supporting Information, the expression of NLRP1 and NLRP3 receptors on the surface of neutrophils was significantly upregulated after PdIr@OMVs treatment. This finding is significant because NLRP1 and NLRP3 are important components of the inflammasome, which plays a key role in regulating immune response and inflammation processes. The upregulation of NLRP1 and NLRP3 suggested that PdIr@OMVs enhanced the immune response of neutrophils by activating these receptors to counter the immunosuppression caused by sepsis.

In vivo immunostimulatory effect of PdIr@OMVs was then systematically investigated on the 24th h of treatment. Firstly, we examined the maturation of DCs in the inguinal lymph nodes of immunocompromised septic mice. As shown in Figure 7f,g, compared to PBS‐treated group, significant enrichment of maturated DCs (44.81% of CD80^+^CD86^+^) was found in lymph nodes after PdIr@OMVs treatment, and NIR irradiation could further enhanced the positive effect of PdIr@OMVs on DCs maturation (49.58% of CD80^+^CD86^+^). In addition, we assessed the differentiation of T cells in the spleen and found that the treatment with PdIr@OMVs greatly promoted the differentiation of T cells (CD3^+^) in the spleen of immunocompromised septic mice (Figures S34 and S35, Supporting Information). In particular, the proportions of cytotoxic T cells (8.84% of CD3^+^CD8^+^) and helper T cells (24.61% of CD3^+^CD4^+^) in PdIr@OMVs + NIR‐treated group were markedly increased (Figure 7h,i). Furthermore, serum concentrations of proinflammatory cytokines of TNF‐α, IL‐6, IL‐1β, IL‐12p40, interleukin‐2 (IL‐2), and interferon‐γ (IFN‐γ) upon treatment with PdIr@OMVs + NIR were efficiently increased compared to the control (Figure 7j and Figure S36, Supporting Information), showing consistent results with those of DCs maturation and T cells differentiation. Moreover, the antibody titer in the serum of immunocompromised septic mice on the 3rd day of treatment was measured to investigate the effect of PdIr@OMVs on humoral immunity. As shown in Figure 7k, the level of antibody toward MDR *E. coli* produced in PdIr@OMVs + NIR‐treated group was two‐fold higher than that of PBS‐treated group, indicating the activated humoral immunity. These encouraging cross‐validated results supported by acceleration and increasement in DCs maturation, T cells differentiation, proinflammatory cytokines secretion, and antibody titer demonstrate that the paralyzed adaptive immunity of immunocompromised septic mice is rapidly and effectively recovered upon PdIr@OMVs treatment.

By collaboratively evoke innate and adaptive immunity, PdIr@OMVs could enable the restoration of systemic immune homeostasis and circumvent organ damage of septic mice. As shown in Figure S37, Supporting Information, the number of WBCs in blood of immunocompromised septic mice upon treatment with PdIr@OMVs + NIR was significantly increased to the normal level on the 3rd day of treatment, indicating a rapid restoration of immune homeostasis. Figure 7l shows the H&E staining images of main organs of immunocompromised septic mice on the 3rd day of treatment. It was found that damage to the lung and kidney in septic mice was featured by severe tissue hyperplasia in perivascular area and inflammatory cell infiltration, which was greatly alleviated in PdIr@OMVs + NIR‐treated group. Collectively, these multi‐dimensional results verify the outstanding in vivo performance of PdIr@OMVs for immunocompromised MDR bacterial sepsis treatment.

### 2.9 In Vivo Therapeutic Efficiency of PdIr@OMVs in an Immunocompromised MDR Polymicrobial Sepsis Model

**FIGURE 8:**
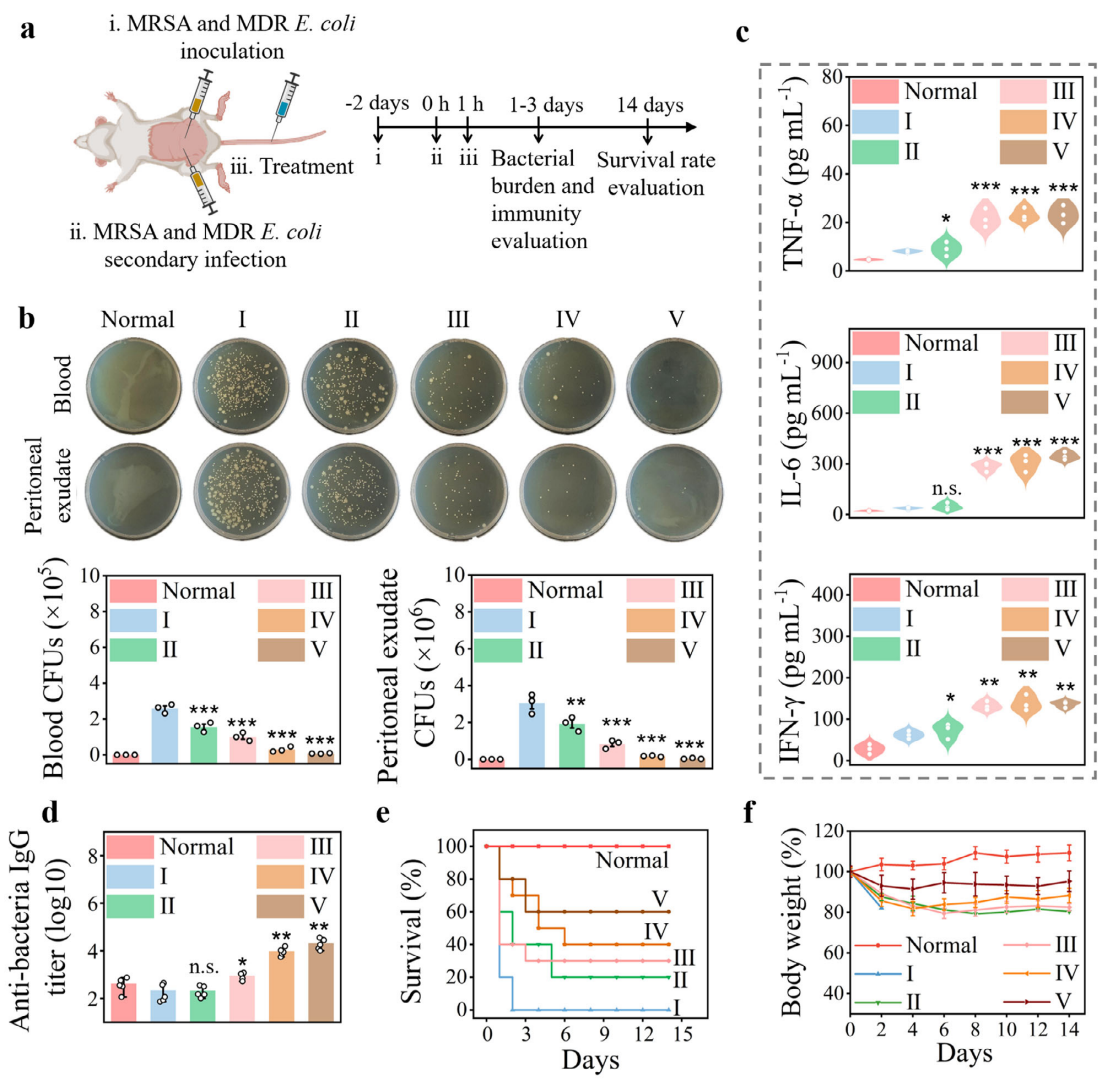
In vivo therapeutic efficiency of PdIr@OMVs in an immunocompromised MDR polymicrobial sepsis model. (a) Experimental timeline for the treatment of immunocompromised MDR polymicrobial septic mice. (b) Agar plate images of bacterial culture extracted from the blood and peritoneal exudate (intracellular) of immunocompromised MDR polymicrobial septic mice on the 24th h of different treatments, and the corresponding quantitative data of bacterial colonies (*n* = 3). (c) The secretion of TNF‐α, IL‐6, and IFN‐γ retrieved from the serum of immunocompromised MDR polymicrobial septic mice on the 24th h of different treatments (*n* = 3). (d) Anti‐bacterial lgG (anti‐MDR *E. coli* lgG plus anti‐MRSA lgG) titer retrieved from the serum of immunocompromised MDR polymicrobial septic mice on the 3rd day of different treatments (*n* = 3). (e, f) Survival rate (e) and body weight (f) of immunocompromised MDR polymicrobial septic mice during the 14 days of different treatments (*n* = 10). Five treatment groups were employed including PBS (I), NIR (II), PdIr (III), PdIr@OMVs (IV), and PdIr@OMVs + NIR (V), and healthy mice was used as the negative control.

In clinical practice, septic patients are usually exposed to mixed bacterial infections, and one‐third of patients who survive hospitalization for sepsis die during the following year due to immunosuppression‐induced inability to resist recurrent infections [5, 55]. Encouraged by the robust performance of PdIr@OMVs in the treatment of MDR *E. coli*‐induced septic mice with immunoparalysis, we further assessed their therapeutic efficiency in a mouse model of immunocompromised MDR polymicrobial sepsis to better mimic the clinical scenario. This mouse model was constructed by first intraperitoneal injection of a mixture of MRSA and MDR *E. coli* to induce polymicrobial sepsis, and the surviving mice progressing to systemic immunosuppressive state, as reflected by a significantly decreased serum TNF‐α level shown in Figure S38, Supporting Information, were further challenged by a secondary polymicrobial infection of MRSA and MDR *E. coli*. The immunocompromised septic mice were allocated into five groups (10 mice per group), and was treated with PBS, NIR, PdIr, PdIr@OMVs, and PdIr@OMVs + NIR at 1 h after secondary polymicrobial inoculation, and healthy mice were used as negative control (Figure 8a). As shown in Figure 8b, treatment with PdIr@OMVs + NIR significantly reduced the bacterial burden in the blood and peritoneal exudate of immunocompromised septic mice compared to PBS‐, NIR‐, PdIr, or PdIr@OMVs‐treated mice. Simultaneously, serum levels of proinflammatory cytokines of TNF‐α, IL‐6, IFN‐γ, IL‐1β, IL‐12p40, and IL‐2 and antibacterial lgG titer in PdIr@OMVs + NIR‐treated mice were greatly enhanced, indicating the activation of systemic immune response (Figure 8c,d, and Figure S39, Supporting Information). Furthermore, serum levels of both proinflammatory cytokine of TNF‐α and anti‐inflammatory cytokine of IL‐10 after treatment with PdIr@OMVs for 3 days were basically the same as those in normal healthy mice, indicating the restoration of immune homoeostasis (Figure S40, Supporting Information). By effectively eradicating bacteria and rapidly restoring immune homoeostasis, treatment with PdIr@OMVs + NIR lead to a substantially enhanced survival rate (60%) and a steady increase in body weight in immunocompromised septic mice (Figure 8e,f). These in vivo results prove the therapeutic efficacy of PdIr@OMVs in an immunocompromised MDR polymicrobial sepsis model, indicating their potential application for clinical intervention of MDR bacterial sepsis and septic shock.

## 3 Discussion

Sepsis has a complex pathophysiology in which both excessive and refractory inflammatory responses are hallmark features, and the development and manifestation of sepsis varies as it transits from an initial acute hyper‐inflammatory phase to a late anti‐inflammatory/immunosuppressive phase. Earlier attempts to treat human sepsis by reducing the acute hyper‐inflammatory reaction uniformly failed, mainly because of the rapid shift of sepsis‐related immune responses from immune activation to immunoparalysis. Emerged evidence has shown that the hypo‐responsive status of the immunoparalysis phase not only prolongs the primary infection and increases the risk of secondary infection by fostering intracellular bacterial growth, but also predicts a worse outcome of sepsis as evidenced by the much higher mortality rate of septic patients in immunoparalysis. Critically, there are no current effective treatments that target the immunoparalysis phase of sepsis, except the use of antibiotics and stabilizing organ functions, which improve survival by approximately 10% only. Therefore, developing effective strategy to tackle sepsis‐induced immunoparalysis is critical for sepsis management to improve the survival in septic patients.

This immunoparalysis phase of sepsis is characterized by intracellular persistent infection and disability of innate and adaptive immune response. Eradicating intracellular infections and reinvigorating systemic innate‐adaptive immune response are critical for tackling sepsis‐induced immunoparalysis, yet effective strategies remain elusive. In this study, we develop a PdIr@OMVs‐based robust strategy for rescuing MDR bacterial sepsis‐induced immunoparalysis via intracellular bacteria elimination and systemic innate‐adaptive immune response orchestration. PdIr@OMVs exhibit unique LSPR‐enhanced POD‐like catalytic activity, and inherited the remarkable immunocyte‐targeting capability and adjuvanticity of biocompatible OMVs. We demonstrate that PdIr@OMVs not only potentiate the phagolysosomal killing effect of impaired macrophages to eradicate intracellular bacteria and burst antigen release, but also allowed rapid activation/maturation of DCs to boost the presentation of bacterial antigen, thus boosting systemic innate‐adaptive immune response during immunoparalysis. In two mouse models of immunocompromised MDR bacterial sepsis, PdIr@OMVs collaboratively reduced bacterial burden and restored immune homeostasis, thereby circumventing organ damage and significantly improving the survival rate of septic mice. In contrast to conventional immunostimulatory therapies, PdIr@OMVs can collaboratively eradicate intracellular MDR bacteria and rescue immunoparalysis, offering a promising therapeutic paradigm for sepsis and septic shock.

## 4 Materials and Methods

### 4.1 Chemicals and Materials

Sodium tetrachloropalladate (Na_2_PdCl_4_), sodium hexachloroiridate hydrate (Na_3_IrCl_6_·*x*H_2_O), L‐ascorbic acid (AA), and polyvinylpyrrolidone (PVP, *M*_w_ = 55,000 Da), cell counting kit‐8 (CCK‐8), cyclophosphamide, 2,7‐dichlorodihydrofluorescein diacetate (DCFH‐DA) were purchased from Sigma‐Aldrich. Ethylene glycol (EG), ethanol, potassium bromide (KBr, 99%), acetone, *N*,*N*‐dimethylformamide (DMF) were obtained from Beyotime Institute of Biotechnology. 3,3',5,5'‐tetramethylbenzidine (TMB), methylene blue (MB), 5,5‐dimethyl‐1‐pyrroline‐*N*‐oxide (DMPO), live/dead bacterial staining agent, and crystal violet were purchased from Thermo Fisher. Hydrogen peroxide assay kit, BCA protein quantification kit and hydroxyl radical detection kit were obtained from Nanjing Jiancheng Bioengineering Research Institute. Mouse ELISA kits of TNF‐α, IL‐1β, IL‐6, IL‐12p40, IL‐10, IL‐2, and IFN‐γ were purchased from Sangon Biotech (Shanghai) Co., Ltd. Mouse lymphocyte isolation solution, and mouse lymphocyte culture medium were obtained from Shenzhen Dakewe Bio‐engineering Co., Ltd. Antibodies were purchased from Biolegend.

### 4.2 Preparation of PdIr@OMVs

Pd nanocubes were first synthesized according to previously reported method [30]. In brief, 105 mg of PVP, 60 mg of AA, and 300 mg of KBr were dissolved in 8 mL of DI water, and preheated at 80°C for 10 min. Next, 3 mL of 19 mg mL^−1^ Na_2_PdC1_4_ was added rapidly to the reaction solution. After 3 h reaction, the Pd nanocubes precipitate was collected by centrifugation (26,000 rpm, 30 min), cleaned by acetone and finally dispersed in EG. PdIr were synthesized based on the method previously reported [31]. First, 100 mg of PVP and 60 mg of AA were dissolved in 10 mL of EG and kept at 100°C for 1 h. 0.6 mL of synthesized Pd nanocubes (2.0 mg mL^−1^) were then introduced to the solution and the reaction temperature was increased to 200°C. Next, 4 mL of Na_3_IrCl_6_·*x*H_2_O (0.5 mg mL^−1^, dissolved in EG) was injected at a rate of 0.5 mL h^−1^ into the above mixture. The reaction was continued for 10 min after the injection was completed, and rapidly terminated by placing the reaction system in an ice‐water bath. The PdIr precipitate was collected by centrifugation (10,000 rpm, 30 min), and finally dispersed in DI water. The doping ratio of Ir element in PdIr was controlled by adjusting the molar mass of input Ir precursor of Na_3_IrCl_6_·*x*H_2_O. PdIr@OMVs were finally prepared by encapsulating synthesized PdIr with OMVs through polycarbonate film extrusion [32, 33]. In brief, 1 mL of synthesized PdIr (2 mg mL^−1^ of Pd element) was ultrasonically mixed with 1 mL of attenuated *E. coli*‐derived OMVs (1 mg mL^−1^) obtained by vacuum filtration and ultracentrifugation for 10 min. The mixed solution was then extracted under the action of a liposome extractor loaded with a polycarbonate membrane of 100 nm to prepare PdIr@OMVs.

### 4.3 DFT Calculation of POD‐Like Reaction of PdIr

In this work, first‐principles calculations were performed using the Vienna ab initio simulation package (VASP) based on DFT [56]. The Perdew–Burke–Ernzerhof (PBE) functional was used to describe the electron‐electron interaction [57]. The electron wave function was modeled using a plane wave with a cutoff energy of 400 eV. To account for van der Waals interactions, Grimme's DFT‐D3 dispersion‐correction approach was applied to all exchange‐correlation energies [58]. A four‐layer supercell was utilized to model Ir(111), Pd(100), and Pd(100)‐Ir (with ten Ir(111) atoms on Pd(100)). To obtain the atomic diffusion pathway and identify transition state (TS) configurations, the climbing image nudged elastic band (CI‐NEB) approach implemented with ab initio DFT was utilized. The adsorption formation energy $E_{\mathrm{ad}}$ is defined as follows:

$$E_{\mathrm{ad}}=E_{\mathrm{total}}-E_{\mathrm{slab}}-E_{H_{2}O}$$

where $E_{\mathrm{total}}$ is the total energy of H_2_O@slab (slab = Ir(111), Pd(100), and Pd(100)‐Ir). $E_{\mathrm{slab}}$ and $E_{H_{2}O}$ is the energy of the surface model and the energy of H_2_O, respectively.

### 4.4 LSPR‐Enhanced POD‐Like Activity of PdIr@OMVs

The performance of PdIr@OMVs to oxidize TMB, degrade MB, and produce ·OH upon NIR irradiation was investigated to demonstrate the LSPR‐enhanced POD‐like activity of PdIr@OMVs. In these experiments, the experimental details of TMB oxidation, MB degradation, and ESR‐based ·OH measurement were exactly the same as those of POD‐like activity investigation described above except NIR laser irradiation (808 nm, 0.8 W cm^−2^, 5 min). To explore the mechanism of LSPR‐enhanced POD‐like activity of PdIr@OMVs, the ability of PdIr@OMVs to oxidize TMB in the presence of water cooling and photo‐induced hole scavenger of EtOH was evaluated. For the water‐cooling assay, PdIr@OMVs (15 µg mL^−1^ of Pd element) was added to the mixture of TMB (3.75 mm) and H_2_O_2_ (150 µm), and the reaction was carried out for 10 min, during which the reaction system was irradiated with an 808 nm laser (0.8 W cm^−2^, 5 min) and the temperature was maintained at 25°C by using a water bath. For TMB oxidation with photo‐induced hole scavenger, the solutions of PdIr@OMVs (15 µg mL^−1^ of Pd element), TMB (3.75 mm) and H_2_O_2_ (150 µm) was mixed and reacted in the presence of EtOH (10 vol%) and NIR irradiation (808 nm, 0.8 W cm^−2^, 5 min).

### 4.5 POD‐Like Reaction Kinetics of PdIr@OMVs

Steady‐state kinetic assay of POD‐like reaction of PdIr@OMVs toward different substrates (TMB and H_2_O_2_) was performed by adding PdIr@OMVs (15 µg mL^−1^ of Pd element) into the mixture of TMB and H_2_O_2_ with different concentrations, and the absorption of the mixture at 652 nm was measured after 10 min of reaction. The steady‐state kinetic parameters were calculated according to the Michaelis–Menten equation:

$$v=V_{\max}\times \left[S\right]/\left(K_{m}+\left[S\right]\right)$$

where ν is the initial velocity of the reaction, *K*_m_ is the Michaelis constant, *V*_max_ is the maximum reaction velocity, and [*S*] is the concentration of substrate [45].

### 4.6 Bacteria Culture and Antimicrobial Performance of PdIr@OMVs

MDR *E. coli* (ATCC BAA‐3049) and MRSA (ATCC 33591) were employed. To perform antimicrobial experiments, 10^7^ CFU of bacteria were co‐incubated with PdIr@OMVs (50 µg mL^−1^ of Pd element) and H_2_O_2_ (100 µm) for 30 min upon NIR laser irradiation (808 nm, 0.8 W cm^−2^, 5 min). The antimicrobial performance was evaluated by the bacterial growth curve analysis, live/dead bacterial staining assay, SEM‐based bacterial morphology investigation, and biofilm eradiation experiments (see the experimental details in Supporting Information).

### 4.7 PdIr@OMVs‐Potentiated Phagolysosomal Killing of Impaired Macrophages In Vitro

To demonstrate the capability of PdIr@OMVs for potentiating the phagolysosomal killing of impaired macrophages, M2 J774A.1 macrophages were secondly infected with 10^6^ CFU of MDR *E. coli* and MRSA (lysozyme was used to eliminate extracellular bacteria), respectively, and the performance of PdIr@OMVs for improving intracellular bacteria elimination, M1 macrophages polarization, and pro‐inflammatory cytokines secretion was evaluated. For intracellular bacteria elimination assay, infected M2 J774A.1 macrophages were incubated with PdIr@OMVs (50 µg mL^−1^ of Pd element) for 24 h upon NIR irradiation (808 nm, 0.8 W cm^−2^, 5 min) and lysed with 1% Triton X‐100, and intracellular bacteria were quantified by bacterial plate counting. For M2/M1 macrophages polarization assay, infected M2 J774A.1 macrophages were incubated with PdIr@OMVs (50 µg mL^−1^ of Pd element) for 24 h upon NIR irradiation (808 nm, 0.8 W cm^−2^, 5 min), and the expression of CD80 (M1 macrophage marker) and CD206 (M2 macrophage marker) were quantified by flow cytometry. For pro‐inflammatory cytokines secretion assay, infected M2 J774A.1 macrophages were incubated with PdIr@OMVs (50 µg mL^−1^ of Pd element) for 24 h upon NIR irradiation (808 nm, 0.8 W cm^−2^, 5 min), and the secretion of pro‐inflammatory cytokine of TNF‐α and anti‐inflammatory cytokine of IL‐10 were quantified by using the corresponding ELISA kits.

### 4.8 PdIr@OMVs‐Promoted DCs Activation and Maturation in Vitro

To evaluate the effect of PdIr@OMVs on the activation and maturation of DCs, immature DCs was stimulated by PdIr@OMVs and the expression of co‐stimulatory molecules and main stimulatory molecules as well as the secretion of pro‐inflammatory cytokines were analyzed. In brief, DC2.4 cells were inoculated in 6‐well plates (10^6^ cells per well) overnight, and then incubated with PdIr@OMVs (50 µg mL^−1^ of Pd element) for 24 h. The supernatants were collected at different time points (4, 8, 12, 24 h) and the secretion of pro‐inflammatory cytokines (TNF‐α, IL‐1β, IL‐6, IL‐12p40) was analyzed by ELISA. At 24 h post‐treatment of PdIr@OMVs, DC2.4 cells were collected via EDTA treatment and centrifugation, and the expression of co‐stimulatory molecules (CD40, CD80, and CD86) and main stimulatory molecule (MHC‐II) was determined based on flow cytometry using the corresponding fluorescent dyes‐labeled antibodies (APC‐CD40 antibody, APC‐CD80 antibody, APC‐CD86 antibody, APC‐I‐A/I‐E antibody). In addition, after 24 h stimulation of PdIr@OMVs, DC2.4 cells were further incubated with the supernatant of MDR *E. coli*‐infected macrophages treated by PdIr@OMVs + NIR (SIMN) for 12 h, and the expression of the co‐stimulatory molecules of CD80 and CD86 was quantified by flow cytometry using the PE‐Cy7 CD80 antibody and APC CD86 antibody, respectively.

### 4.9 Construction of Mouse Model of MDR *E. Coli*‐Induced Sepsis With Immunoparalysis

All experiments conducted in this study were reviewed and approved by the Ethical Committee of Soochow University (Approval No. SUDA 20231228A01). Female mice (BALB/c, 6 weeks) were employed and allowed to adapt in the laboratory for 1 week before experiment. The mouse model of MDR *E. coli*‐induced sepsis with immunoparalysis was constructed by first administrating the immunosuppressive agent of cyclophosphamide (150 mg kg^−1^) for three consecutive days to cause systemic immunosuppression, and then inoculating lethal doses of 2 × 10^8^ CFU of MDR *E. coli* through intraperitoneal (i.p.) injection.

### 4.10 PdIr@OMVs Rescue MDR *E. Coli*‐Induced Septic Mice With Immunoparalysis

To perform in vivo treatment, immunocompromised septic mice were administrated with PdIr@OMVs (50 µg mL^−1^ of Pd element) 1 h after bacterial inoculation via i.v. injection. Five treatment groups (10 mice per group) were divided including PBS (positive control), NIR (808 nm, 0.8 W cm^−2^, 5 min), PdIr, PdIr@OMVs, and PdIr@OMVs + NIR (808 nm, 0.8 W cm^−2^, 5 min), and the group of normal healthy mice was used as the negative control. At the 24th h of treatment, bacterial burden in blood and peritoneal exudate, enrichment of maturated DCs (CD80^+^CD86^+^) in lymph nodes, the proportion of cytotoxic T cells (CD3^+^CD8^+^) and helper T cells (CD3^+^CD4^+^) in spleen, and the amount of typical proinflammatory factors (TNF‐α, IL‐6, IL‐1β, IL‐12p40, IL‐2, and IFN‐γ) were measured and analyzed. At the 3rd day of treatment, the serum level of anti‐MDR *E. coli* lgG titer, the number of WBCs in serum, and the histological condition of main organs were evaluated. Additionally, the survival and body weight of immunocompromised septic mice during treatment were quantitatively analyzed.

### 4.11 Construction of Immunocompromised Mouse Model of MDR Polymicrobial Sepsis

BALB/c mice were first intraperitoneally injected with 0.1 mL of a mixed suspension of MRSA (2 × 10^8^ CFU) and MDR *E. coli* (2 × 10^8^ CFU), and the surviving mice progressing to systemic immunosuppressive state were then exposed to secondary polymicrobial infection (10^7^ CFU of MRSA and MDR *E. coli*) to induce MDR polymicrobial sepsis with immunoparalysis.

### 4.12 In Vivo Therapeutic Efficiency of PdIr@OMVs in the Immunocompromised MDR Polymicrobial Sepsis Model

To perform in vivo treatment, immunocompromised septic mice were administrated with PdIr@OMVs (50 µg mL^−1^ of Pd element) via i.v. injection 1 h after secondary polymicrobial infection. Five treatment groups (10 mice per group) were divided including PBS (positive control), NIR (808 nm, 0.8 W cm^−2^, 5 min), PdIr, PdIr@OMVs, and PdIr@OMVs + NIR (808 nm, 0.8 W cm^−2^, 5 min), and the negative control of healthy mice. At the 24th h of treatment, bacterial burden in blood and peritoneal exudate, and the levels of typical proinflammatory factors were measured and analyzed. At the 3rd day of treatment, the serum level of anti‐bacterial lgG (anti‐MDR *E. coli* lgG plus anti‐MRSA lgG) titer, and the serum level of TNF‐α and IL‐10 were measured. Additionally, the survival and body weight of immunocompromised septic mice during treatment were quantitatively analyzed.

## Author Contributions

X. Du, Z. Dong and Y. Yan contributed equally to this work. H. Zhou, J. Wang and Y. Li designed experiments and conceived the manuscript. X. Du, Z. Dong and Y. Yan performed most of the experiments. M. Yuan contributed to theoretical calculations. Y. Gong, C. Ma, L. Xu, M. Qu, P. Pan, W. Li, X. Liu and W. Hao assisted in performing in vivo experiments. M. Zhao, Z. Bai, JH. Wang and J. Wang assisted in manuscript writing. Y. Li and H. Zhou directed the project.

## Ethics Statement

All animal experiments were carried out in compliance with the protocols approved by the Ethical Committee of Soochow University (Approval No. SUDA 20231228A01).

## Conflicts of Interest

The authors declare no conflicts of interest.

## Supporting information




**Supplementary File 1**: exp270056‐sup‐0001‐SupMat.docx

## Data Availability

All data needed to support the findings of this study are present in the paper and/or in the Supporting Information. Additional data related to this paper are available from the corresponding authors.
