# Supplementary material for: Immunomodulatory Nanozymes Eradicate Intracellular Infections and Rescue Immunoparalysis for Treating Multidrug‐Resistant Bacterial Sepsis
Source: Exploration (Beijing). 2025 Jun 1;5(5):20250127. doi: 10.1002/EXP.20250127 (PMC12561197; doi:10.1002/EXP.20250127)

Supporting Information

Immunomodulatory nanozymes eradicate intracellular infections and rescue immunoparalysis for treating multidrug-resistant bacterial sepsis

Xuancheng Du^1,2†^, Zhenzhen Dong^1†^, Yan Yan^3†^, Yuan Gong^1^, Meili Yuan^2^, Chengtai Ma^3^, Lingqi Xu^1^, Yuanyuan Qu^2^, Minhan Qu^1^, Peng Pan^1^, Weifeng Li^2^, Xiangdong Liu^2^, Wenyan Hao^1^, Mingwen Zhao^2^, Zhenjiang Bai^1^, Jiang Huai Wang^4^, Jian Wang^1*^, Yong-Qiang Li^2*^ and Huiting Zhou^1*^

^1^ Institute of Pediatric Research, Children’s Hospital of Soochow University, Suzhou 215025, China.

^2^ Institute of Advanced Interdisciplinary Science, School of Physics, Shandong University, Jinan 250100, China.

^3^ Department of Critical Care Medicine, Renmin Hospital of Wuhan University, Wuhan 430060, China.

^4^ Department of Academic Surgery, University College Cork, Cork University Hospital, Cork, Ireland.

**Experimental section**

**Characterization**

The morphology and size of PdIr and PdIr@OMVs were observed by transmission electron microscope (TEM) and high-resolution transmission electron microscope (HRTEM), while the hydrodynamic size and zeta potential were monitored by Nanosizer. The UV-vis absorption spectrum of PdIr was characterized by UV-vis spectrophotometer. The X-ray diffraction pattern and X-ray photoelectron spectrum of PdIr were recorded by *in-situ* powder X-ray diffractometer (XRD) and photoelectron spectroscopy (XPS), respectively. The concentrations of Pd and Ir element in PdIr were quantified by inductively coupled plasma mass spectrometry (ICP-MS). The protein composition profiles of OMVs and PdIr@OMVs were analyzed by sodium dodecyl sulfate-polyacrylamide gel electrophoresis (SDS-PAGE), and the protein concentrations of OMVs and PdIr@OMVs were measured based on bicinchoninic acid (BCA) assay.

**POD-like activity of PdIr@OMVs**

The POD-like activity of PdIr@OMVs was investigated by TMB oxidation, MB degradation, and ESR-based ·OH measurement. For TMB oxidation, PdIr@OMVs (15 μg mL^-1^ of Pd element) was added to the solution of TMB (3.75 mM) and H_2_O_2_ (150 μM), and the absorption of the mixture at 652 nm was measured after 10 min of reaction to evaluate the oxidation degree of TMB. For MB degradation, PdIr@OMVs (5 μg mL^-1^ of Pd element) was added into the solution of MB (8 μg mL^-1^) and H_2_O_2_ (100 μM), and the absorption of the mixture at 665 nm was recorded after 30 min of reaction to assess the degradation degree of MB. For ESR-based ·OH measurement, PdIr@OMVs (5 μg mL^-1^ of Pd element) was added to the solution of trapping agent of DMPO (25 mM) and H_2_O_2_ (100 μM), and the ESR signal of the mixture was recorded after 30 min of reaction to indicate the generation of ·OH measurement.

**Bacterial growth curve analysis**

10^7^ CFU of bacteria (MDR *E. coli*) was mixed with PdIr@OMVs (50 μg mL^-1^ of Pd element) and H_2_O_2_ (100 μM) for 30 min upon NIR laser irradiation (808 nm, 0.8 W cm^-2^, 5min). The OD_600_ value of the mixed solutions was measured per 1 h to plot the bacterial growth curve within 12 h.

**Live/dead bacterial staining assay**

Live/dead staining assay was used to evaluate the viability of bacteria. In brief, bacteria before and after PdIr@OMVs treatment were mixed with the dye solution containing SYTO 9 and propidium iodide for 30 min in the dark, and then imaged with a confocal fluorescence microscope. Live bacteria were stained by SYTO 9 with green color, while dead bacteria were stained by propidium iodide with red color due to the damage of cell membrane and wall.

**SEM-based morphological study of bacteria**

The morphology of bacteria was characterized by field-emission scanning electron microscopy (FESEM). In brief, the bacteria before and after PdIr@OMVs treatment were fixed with glutaraldehyde (2.5%) in the dark for 2 h, and dehydrated by ethanol solution with different concentrations (50%, 70%, 90% and 100%) for 10 min. The dehydrated bacterial samples were dropped on silicon wafer, and imaged with FESEM after nitrogen drying and platinum coating.

**Biofilm eradication**

The capability of PdIr@OMVs for biofilm formation inhibition and destruction was investigated by crystal violet staining. For biofilm formation inhibition, 10^7^ CFU of MDR *E. coli* suspensions were mixed with PdIr@OMVs (50 μg mL^-1^ of Pd element) in 96-well plates, and then irradicated by 808 nm laser (0.8 W cm^-2^) for 5 min. After 2 days incubation, the plates were gently washed by PBS, and crystal violet ethanol solution was added and reacted for 15 min. The plates after crystal violet staining were then imaged by camera, and the corresponding absorbance of staining solution at 590 nm was measured to indicate the extent of biofilm formation. For mature biofilm destruction, 10^7^ CFU of MDR *E. coli* suspensions were added into 96-well plates and grown 2 days to form integrated biofilm. Then PdIr@OMVs (50 μg mL^-1^ of Pd element) was added onto the surface of mature MDR *E. coli* biofilm followed by laser irradiation (808 nm, 0.8 W cm^-2^, 5 min), and then crystal violet ethanol solution was added and reacted for 15 min. Finally, the plates after crystal violet staining were imaged by camera, and the corresponding absorbance of staining solution at 590 nm was measured to indicate the extent of biofilm destruction.

**Determination of ROS level in bacteria and infected macrophages**

2ʹ,7ʹ-Dichlorofluorescin Diacetate (DCFH-DA) was used to determine the level of ROS in bacteria and infected macrophages. In brief, bacteria and infected macrophages before and after PdIr@OMVs treatment were first incubated with DCFH-DA (10 μM) for 30 min in dark, respectively, and washed three times with PBS after centrifugation. Then bacteria were imaged by a confocal fluorescence microscope, and their fluorescence spectrums were recorded using a microplate reader.

**Cellular uptake of PdIr@OMVs**

To investigate the cellular uptake of PdIr@OMVs in macrophages and normal cells. J774A.1 macrophages and HUVEC cells were incubated with Cy5.5-modified PdIr@OMVs (50 μg mL^-1^ of Pd element) for 2 h, respectively, and imaged using laser confocal fluorescence microscopy. The fluorescence intensities of Cy5.5 in both J774A.1 macrophages and HUVEC cells were quantified and compared to indicate the high-efficiency cellular uptake of PdIr@OMVs in macrophages. The Cy5.5-modified PdIr (50 μg mL^-1^ of Pd element) was used as the control.

**Co-localization assay of PdIr@OMVs and lysosome in macrophages**

J774A.1 macrophages were incubated with Cy5.5-modified PdIr@OMVs (50 μg mL^-1^ of Pd element) for 2 h, and then exposed to LysoTracker Green for 30 min. Subsequently, 1 mL of 4% paraformaldehyde was added to fix J774A.1 macrophages and fluorescent probe of DAPI was employed to stain the nuclei. Finally, J774A.1 macrophages were imaged by laser confocal fluorescence microscopy to assess the co-localization of PdIr@OMVs and lysosomes.

**Construction of M2 macrophages**

M2 macrophages were constructed by bacteria infection to simulate the impaired macrophages in immunosuppression. In brief, J774A.1 macrophages were inoculated in 6-well plates (2×10^5^ cells per well) overnight, and then incubated with 5×10^6^ CFU of FITC-loaded MDR *E. coli* bacteria. The medium was removed after 2 h incubation and extracellular bacteria were eliminated by lysozyme (5 μg mL^-1^) to construct M2 J774A.1 macrophages.

***In vitro* biocompatibility of PdIr@OMVs**

The *in vitro* biocompatibility of PdIr@OMVs was determined by MTT assay using human umbilical vein endothelial cells (HUVEC), mouse monocyte macrophages (J774A.1), and mouse dendritic cells (DC2.4), respectively. In brief, HUVEC, J774A.1, and DC2.4 cells were seeded into a 96-well plate (8000-10000 cells per well) and cultured overnight, respectively. Then the cells were treated by PdIr@OMVs with different concentrations (0, 2, 5, 10, 20, and 50 µg mL^-1^ of Pd element). After 24 h of culture, MTT reagent was added and the cell viability was evaluated with a microplate reader.

**Hemolysis assay**

Fresh red blood cells (RBC) were separated by centrifuge at 3000 rpm for 10 min, washed with cold sterile PBS (pH = 7.4) until the supernatant colorless, and diluted to final concentration (10 %, v/v_0_). Different concentrations (5, 10, 20, 50, 100 µg mL^-1^ of Pd element) of PdIr@OMVs were added to 450 μL of RBCs and incubated at 37 ℃ for 3 h. At the end of incubation, the solution was centrifuged at 3000 rpm for 10 min and the hemolysis rate was determined *via* recording the absorbance at 540 nm. DI water and PBS buffer were used as the positive and negative control, respectively.

***In vivo* biocompatibility of PdIr@OMVs**

Pharmacokinetic distribution, organ pathological examination as well as blood biochemistry and blood routine assays were conducted to evaluate the *in vivo* biosafety of PdIr@OMVs. In brief, PdIr@OMVs (50 μg mL^-1^ of Pd element) was intravenously injected into healthy mice, irradiated with 808 nm laser (0.8 W cm^-2^) for 5 min, and the amount of Pd and Ir element in organs at different time points (24, 48, and 72 h) after PdIr@OMVs injection was determined by ICP-MS to investigate the pharmacokinetic distribution of PdIr@OMVs *in vivo*. In addition, major organs and blood samples were collected on the 7^th^ day of PdIr@OMVs post-injection. HE staining of organs, blood biochemistry and blood routine assays were performed to demonstrate the *in vivo* biocompatibility of PdIr@OMVs. Organ pathological examination, blood biochemistry and blood routine assays of healthy mice injected with PBS were used as the control.

**Statistical analysis**

In our experiments, data were expressed as mean ± standard deviation. When the number of experimental groups was greater than two, differences between groups were performed by one-way ANOVA and Bonferroni's multiple comparison test. When the number of experimental groups was less than or equal to two, differences between groups were detected by student's two-tailed t test. n.s. indicates P > 0.05, * indicates P < 0.05, ** indicates P < 0.01, and *** indicates P < 0.001.

**Supplementary figures**


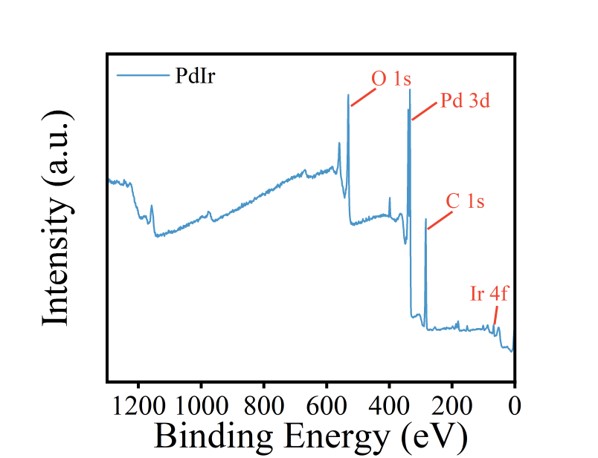


Figure S1 X-ray photoelectron spectrum (XPS) of PdIr nanocatalysts.


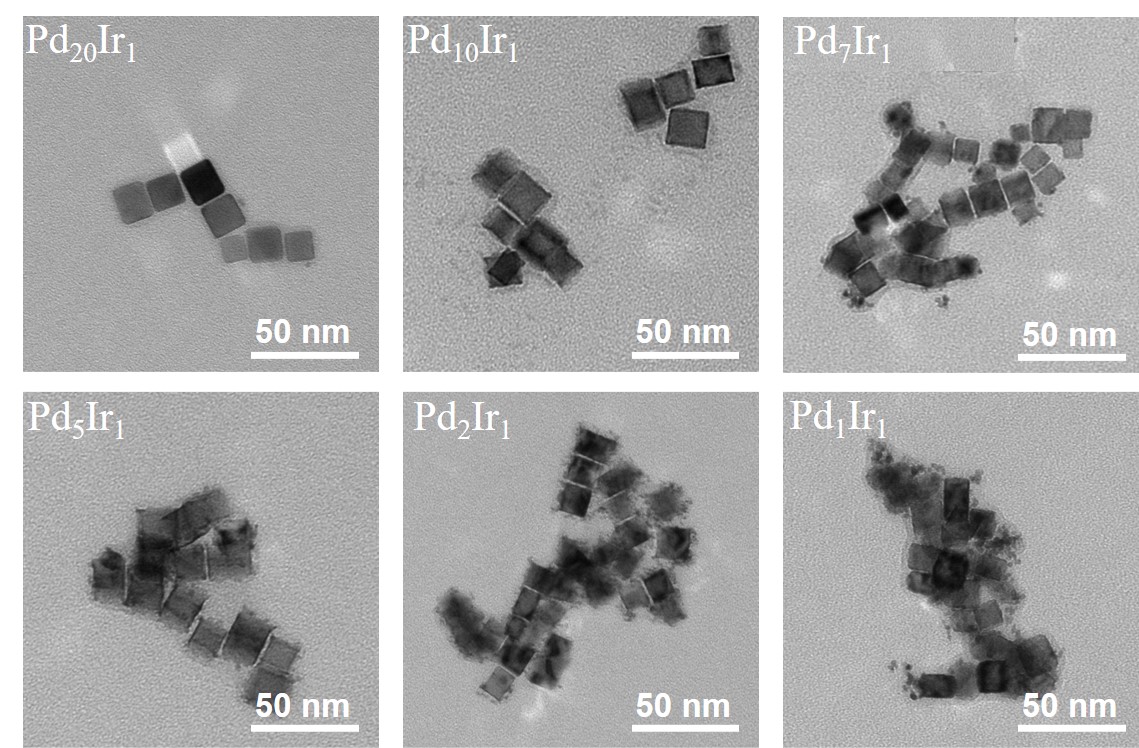


Figure S2 Transmission electron microscopy (TEM) images of PdIr nanocatalysts with different molar ratios of Pd and Ir elements.

**
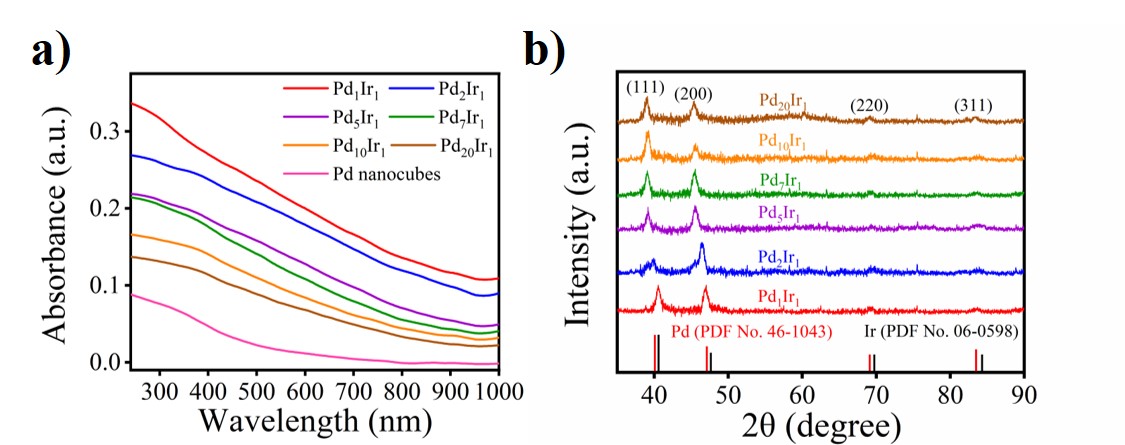
Figure S3** (a) UV-Vis-NIR absorption spectra, and (b) X-ray power diffraction (XRD) patterns of PdIr nanocatalysts with different molar ratios of Pd and Ir elements.


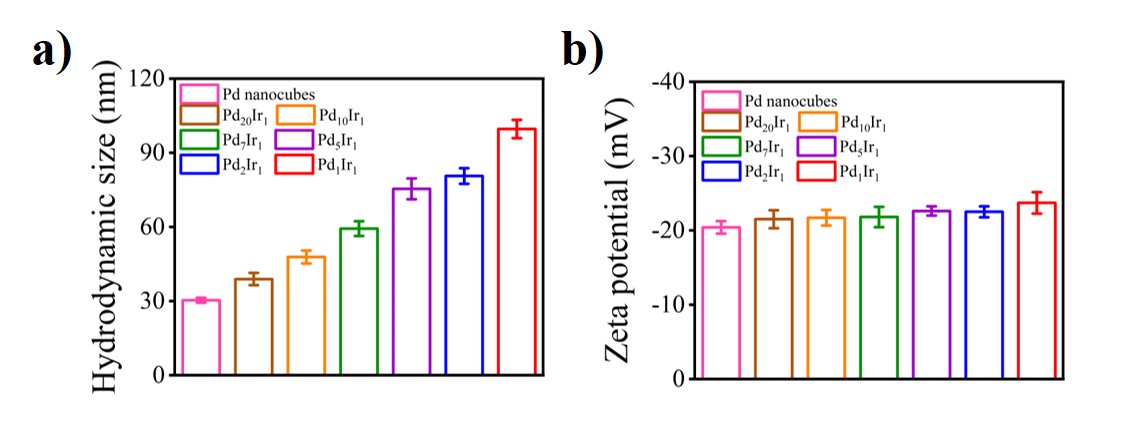


Figure S4 (a) Hydrodynamic diameters, and (b) zeta potentials of PdIr nanocatalysts with different molar ratios of Pd and Ir elements. The values of hydrodynamic size and zeta potential represent the mean of three independent experiments, and the error bars indicate the standard deviation (SD) from the mean.


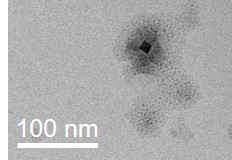


Figure S5 TEM image of PdIr@OMVs.


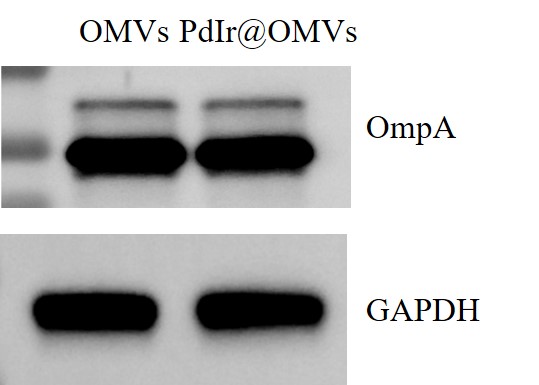


Figure S6 Western blotting analysis for typical protein maker of OmpA in OMVs and PdIr@OMVs.


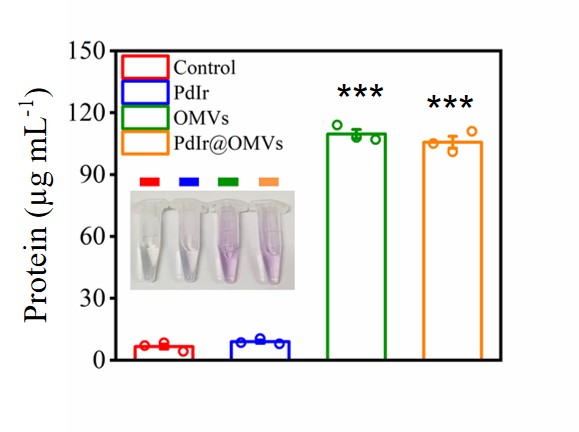


Figure S7 Quantitative protein concentration in PdIr, OMVs, and PdIr@OMVs calculated by BCA assay. The inset shows the corresponding photograph of BCA test solution (bicinchoninic acid, CuSO_4_) in the presence of PdIr, OMVs, and PdIr@OMVs, respectively. The values of protein concentration represent the mean of three independent experiments, and the error bars indicate the SD from the mean. The group of PBS was used as the control. * indicates the contrasts between experimental groups and control. ***P < 0.001.


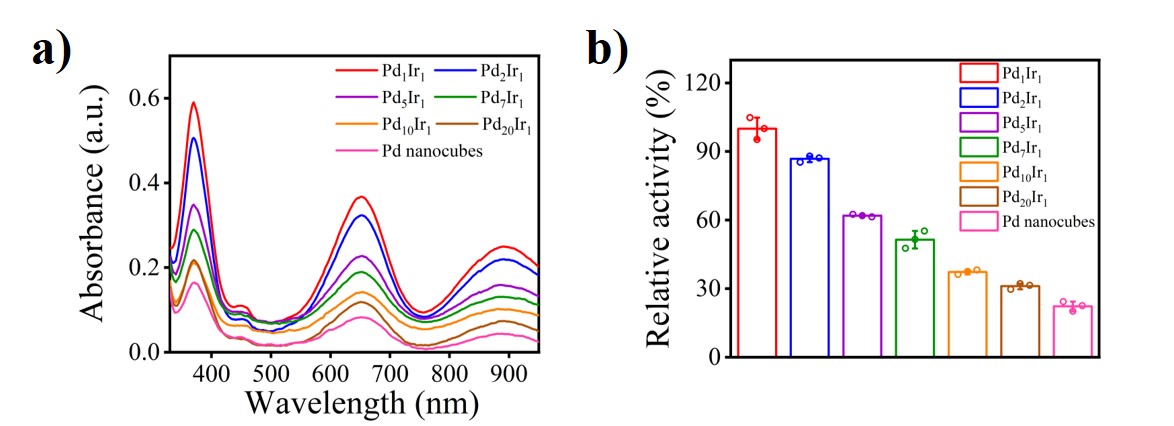


Figure S8 (a) Oxdation of TMB (reaction system: TMB + H_2_O_2_) in the group of PdIr with different molar ratios of Pd and Ir elements. (b) The relative POD-like activity of PdIr with different molar ratios of Pd and Ir elements. The values of relative activity represent the mean of three independent experiments, and the error bars indicate the SD from the mean.


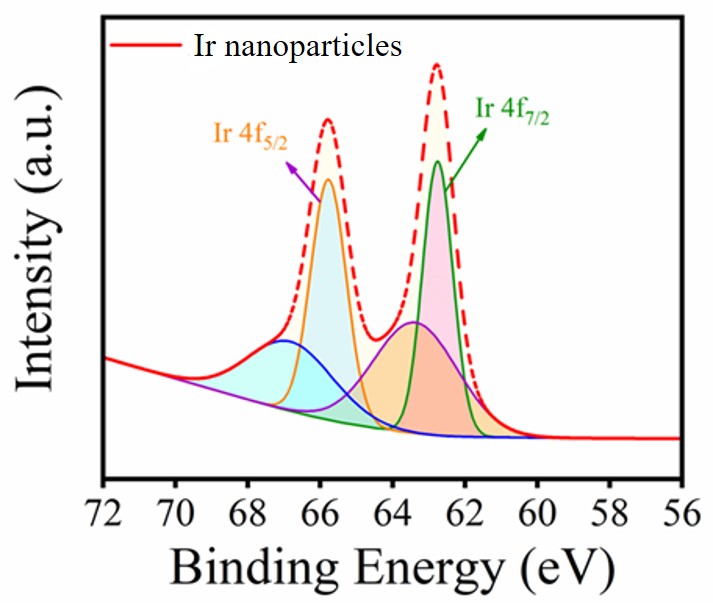


**Figure S9** XPS spectrum of Ir nanoparticles.


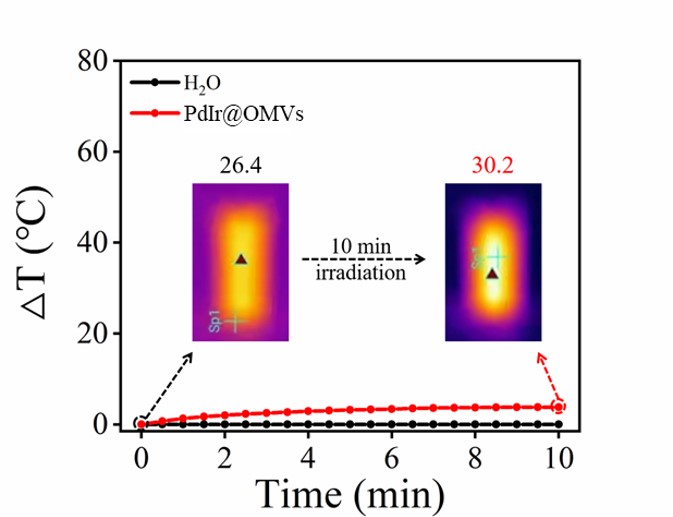


**Figure S10** Photothermal heating curve of PdIr@OMVs under NIR irradiation for 10 min. The group of H2O was used as the control. The inset shows the corresponding photothermal photographs of PdIr@OMVs solution before and after NIR irradiation.


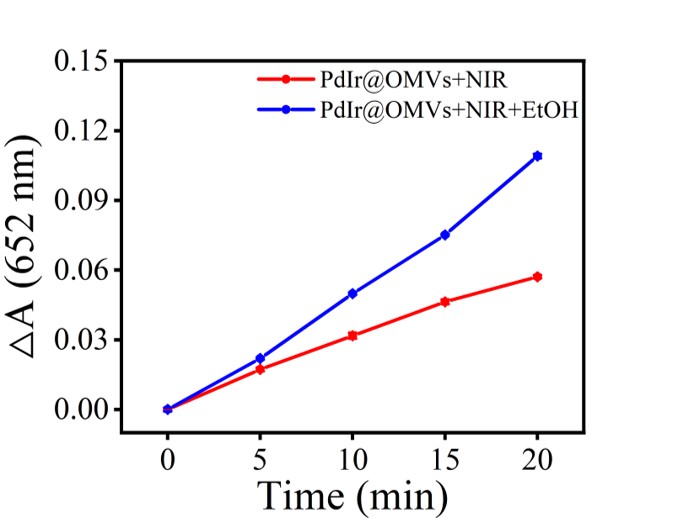


Figure S11 Time-dependent PdIr@OMVs-catalyzed TMB oxidation upon NIR irradiation in the absence/presence of EtOH. The POD-like activity of PdIr@OMVs can oxidize TMB into blue oxTMB with characteristic absorption peak at 652 nm, in the presence of H_2_O_2_.


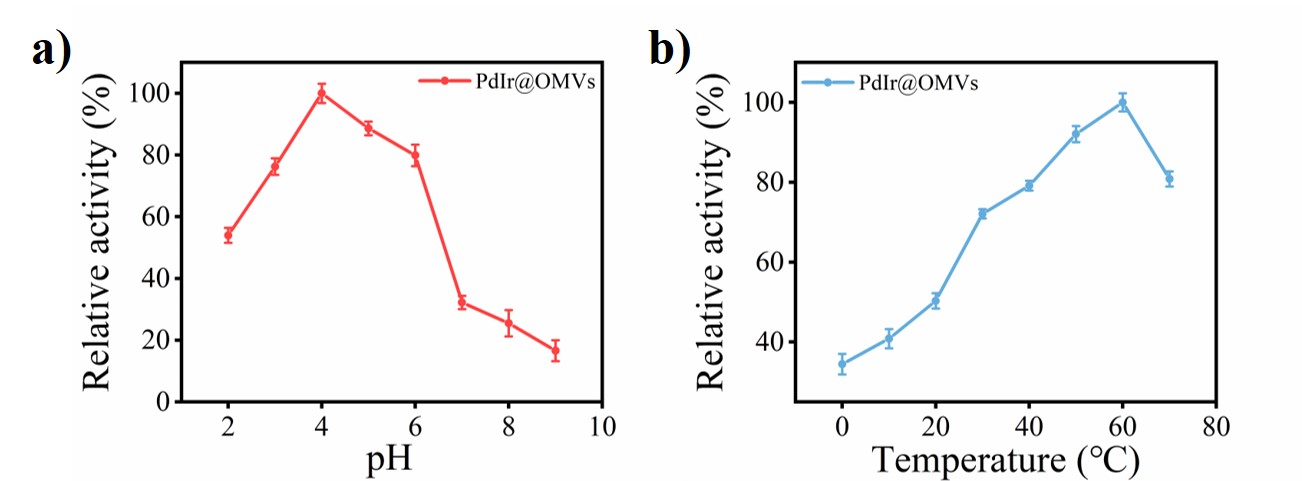


Figure S12 The influence of pH (a) and temperature (b) on the POD-like activity of PdIr@OMVs. The POD-like activity of PdIr@OMVs was determined based on the TMB oxidation experiments. The values of relative activity represent the mean of three independent experiments, and the error bars indicate the SD from the mean.


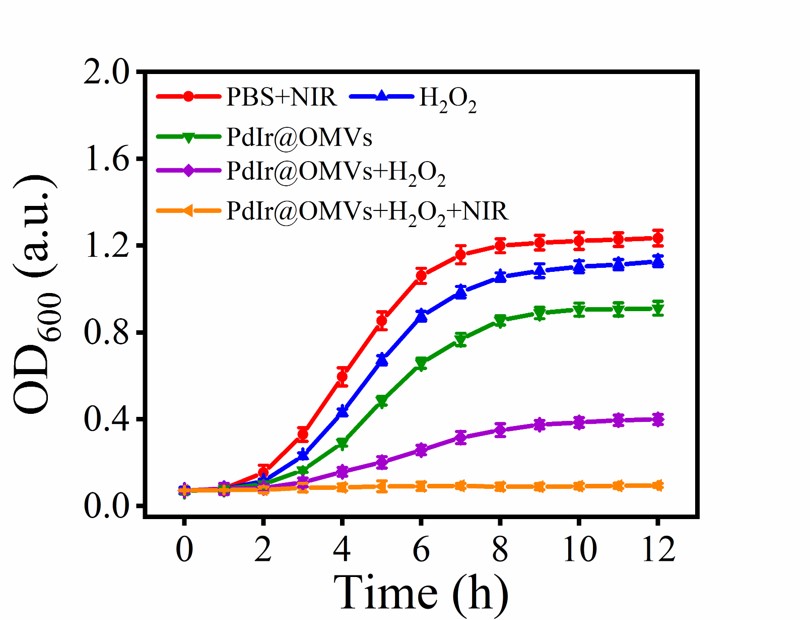


Figure S13 The growth curves of MDR *E. coli* in different treatment groups. The values of OD_600_ represent the mean of three independent experiments, and the error bars indicate the SD from the mean.


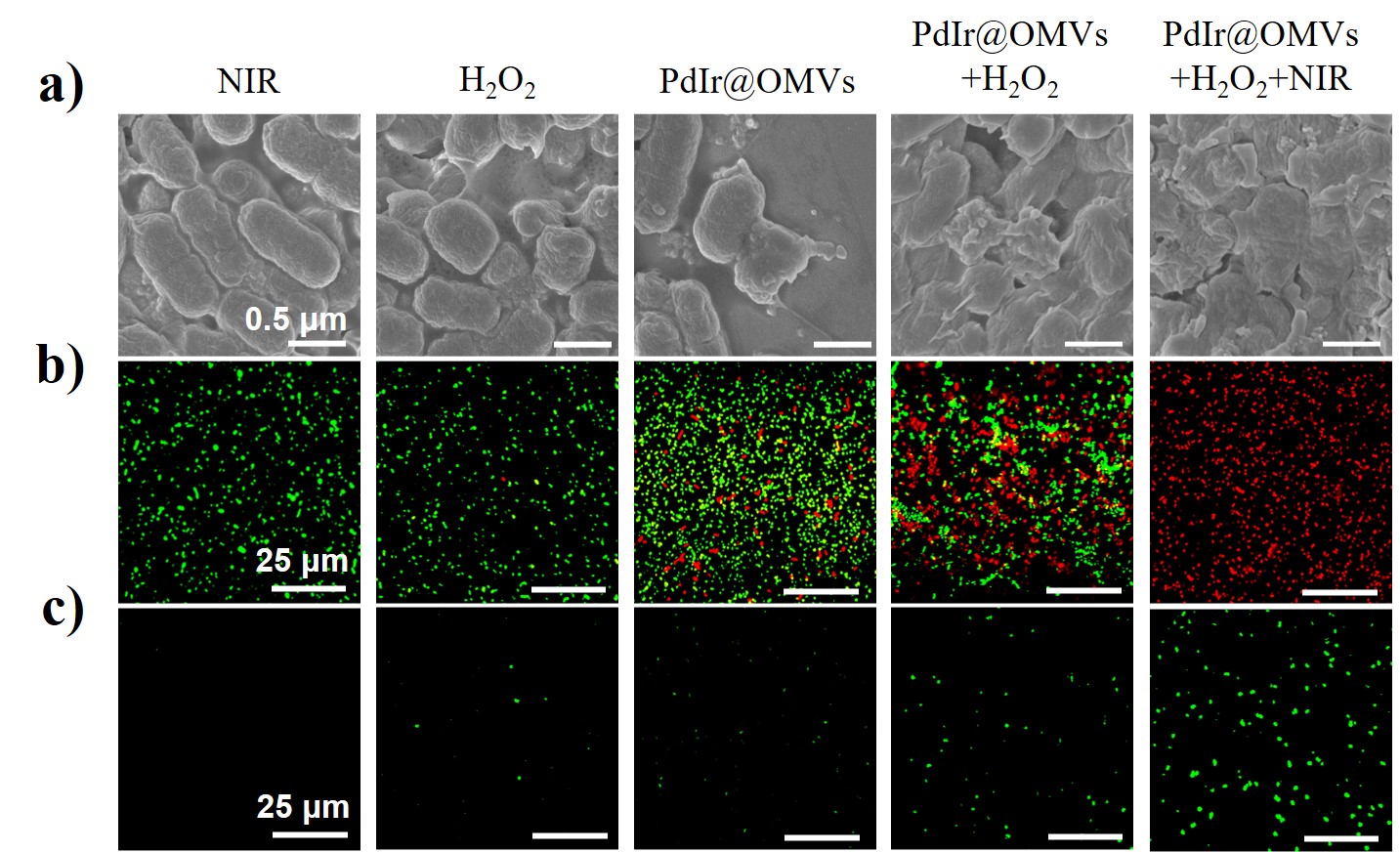


Figure S14 SEM (a), live/dead staining (b), and intracellular ROS staining (c) images of MDR *E. coli* in different treatment groups.


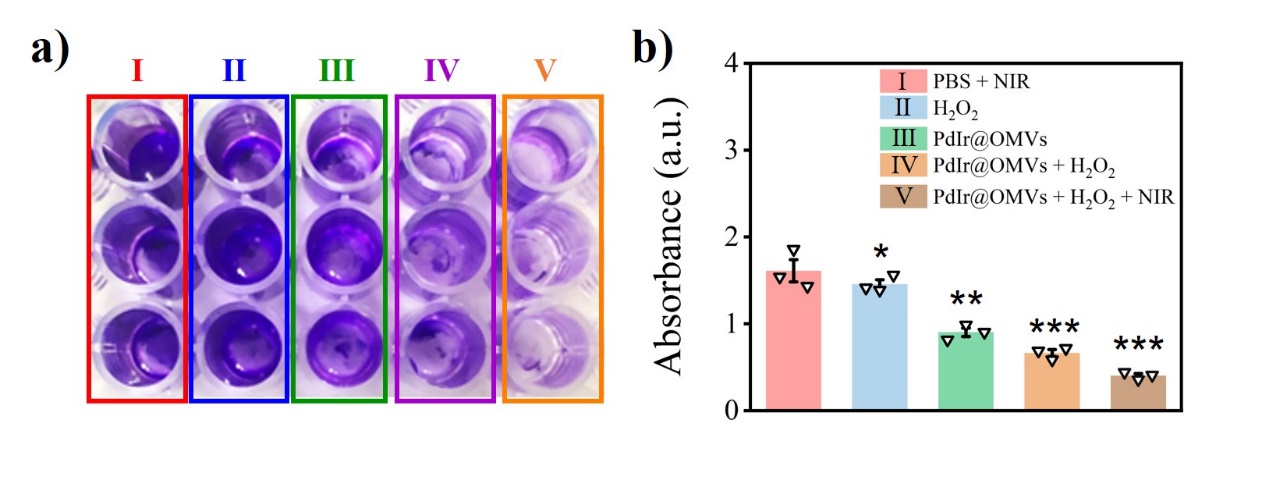


Figure S15 Crystal violet staining images (a) and its corresponding absorbance (b) for the formation of MDR *E. coli* biofilm in different treatment groups. The values of crystal violet absorbance represent the mean of three independent experiments, and the error bars indicate the SD from the mean. The group of PBS plus NIR irradiation (PBS + NIR) was used as the control. * indicates the contrasts between experimental groups and control. ^*^*P* < 0.05, ^**^*P* < 0.01, and ^***^*P* < 0.001.


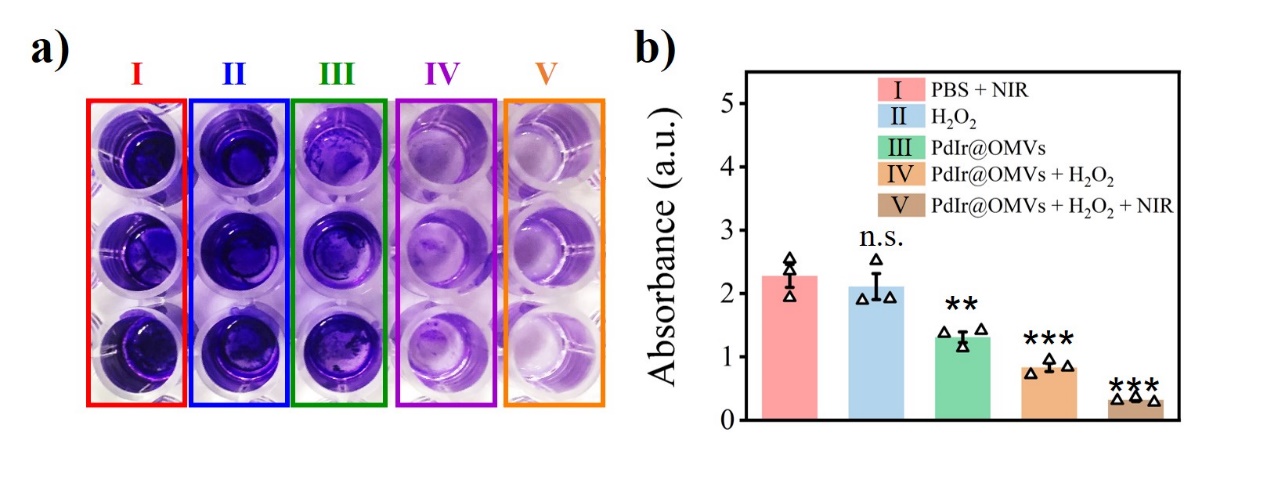


Figure S16 Crystal violet staining images (a) and its corresponding absorbance (b) for integrated MDR *E. coli* biofilm in different treatment groups. The values of crystal violet absorbance represent the mean of three independent experiments, and the error bars indicate the SD from the mean. The group of PBS plus NIR irradiation (PBS + NIR) was used as the control. * indicates the contrasts between experimental groups and control. ^**^*P* < 0.01, ^***^*P* < 0.001, and ^n.s.^*P* > 0.05.


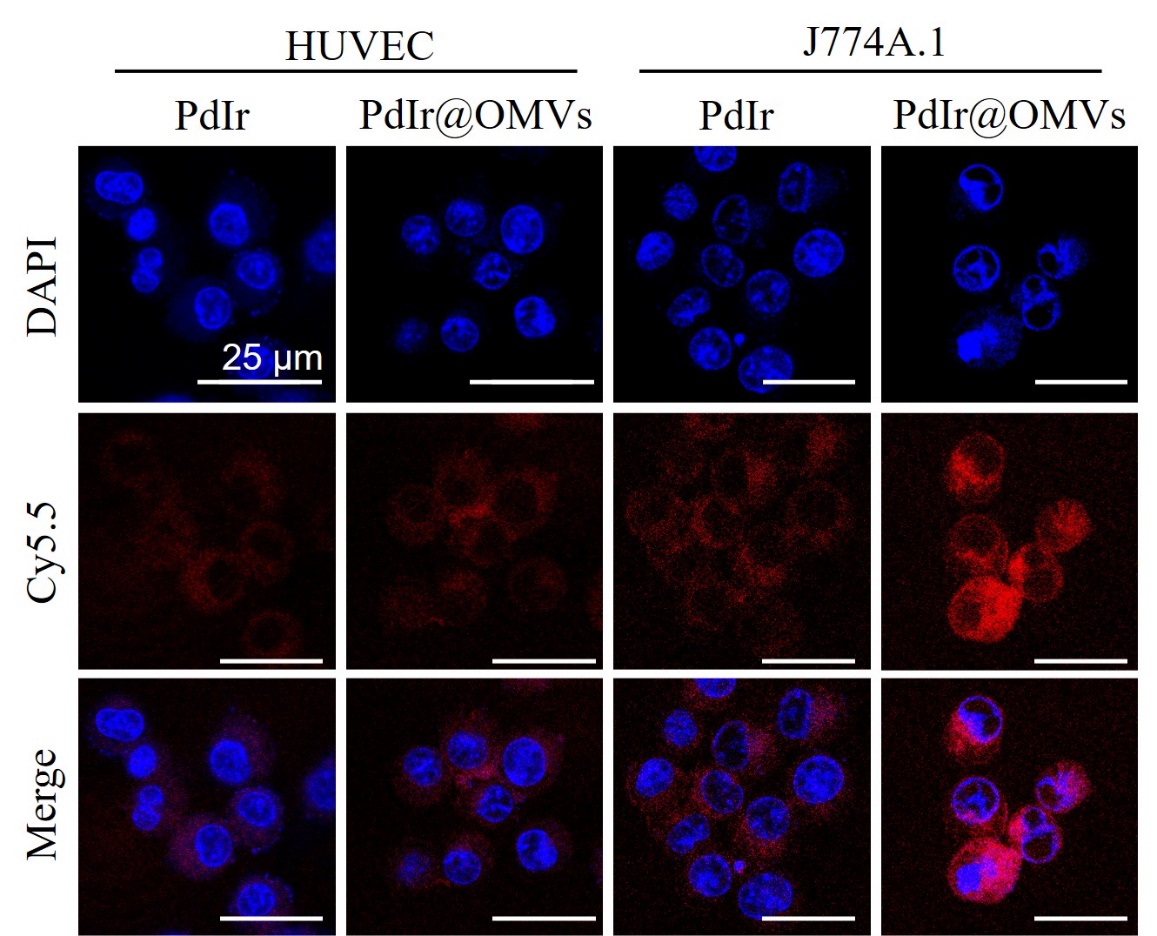


Figure S17 Representative confocal fluorescence images of J774A.1 macrophages and HUVEC cells incubated with Cy5.5-labeled PdIr and Cy5.5-labeled PdIr@OMVs for 4 h, respectively. Fluorescent dye of DAPI with blue color was used to stain cell nucleus.


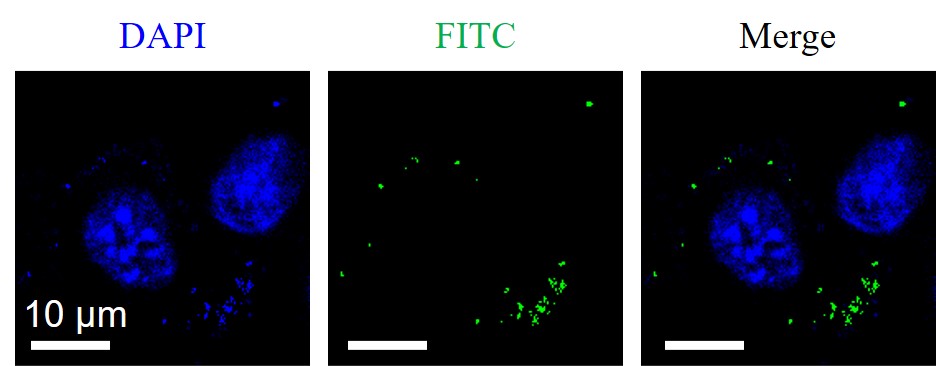


Figure S18 J774A.1 macrophages was infected by FITC-labeled MDR *E. coli* to construct M2 J774A.1 macrophages. Fluorescent dye of DAPI with blue color was used to stain cell nucleus.


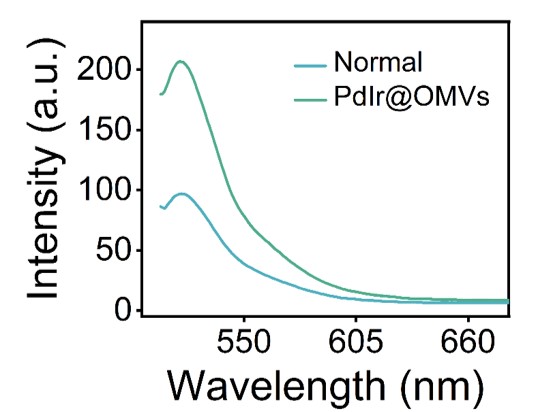


**Figure S19.** Intracellular •OH level in macrophages upon PdIr@OMVs + NIR treatment using specific fluorescent probes O27.


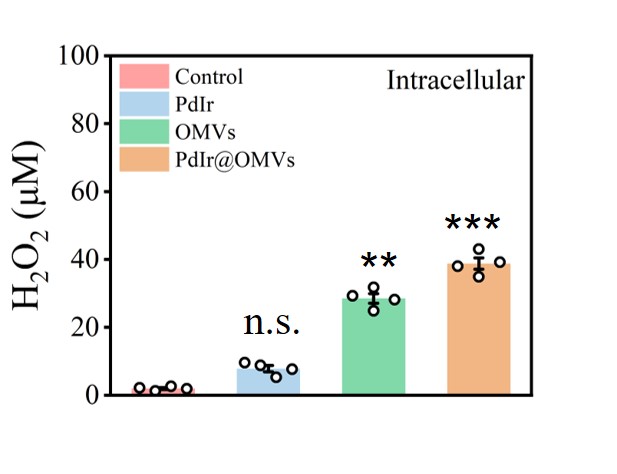


Figure S20 Intracellular H_2_O_2_ level of MDR *E. coli*-infected M2 J774A.1 macrophages in different treatment groups. The group of PBS was used the control. The values of H_2_O_2_ concentration represent the mean of three independent experiments, and the error bars indicate the SD from the mean. * indicates the contrasts between experimental groups and control. ^**^*P* < 0.01, ^***^*P* < 0.001, and ^n.s.^*P* > 0.05.


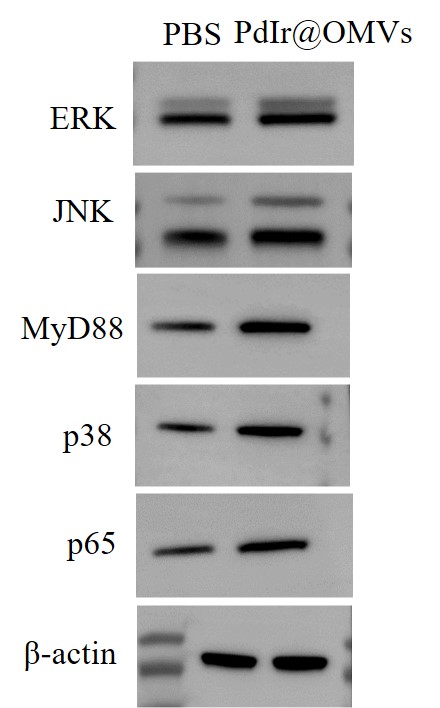


Figure S21 Western blotting analysis for the key proteins of NF-κB and MAPK pathways in macrophages treated by PdIr@OMVs.


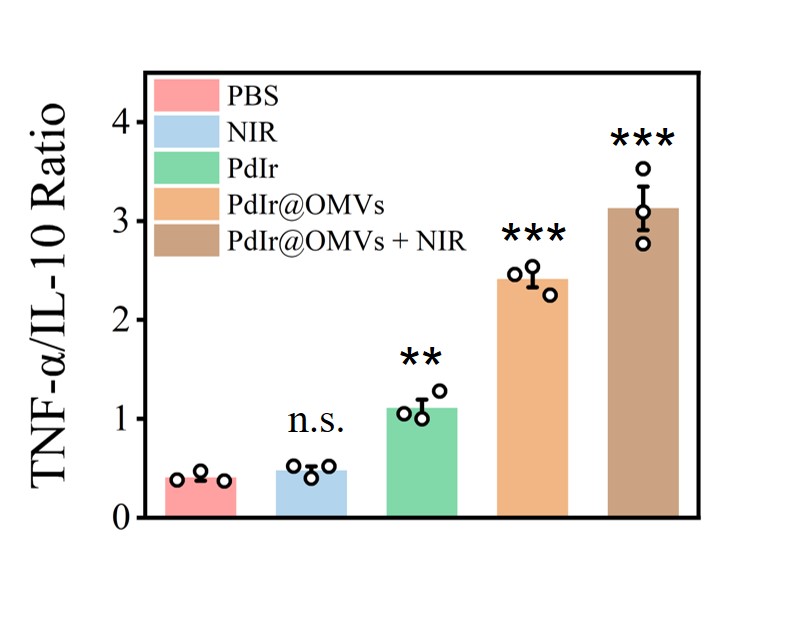


Figure S22 The ratio of TNF-α to L-10 secreted by the MDR *E. coli*-infected M2 J774A.1 macrophages in different treatment groups. The values of TNF-α/IL-10 ratio represent the mean of three independent experiments, and the error bars indicate the SD from the mean. * indicates the contrasts between experimental groups and control (PBS). ^**^*P* < 0.01, ^***^*P* < 0.001, and ^n.s.^*P* > 0.05.


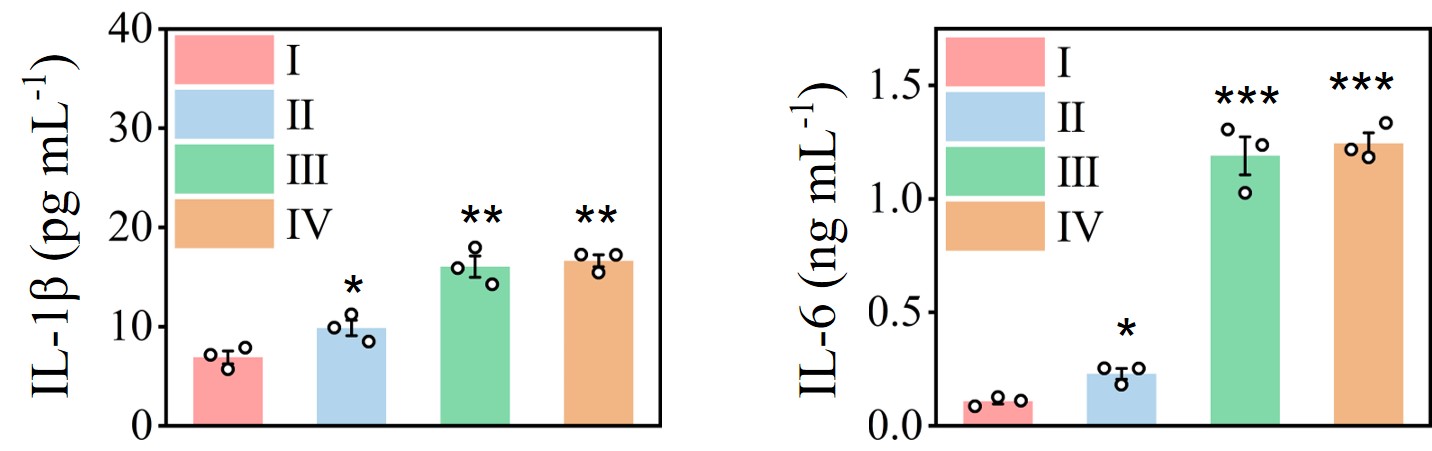


**Figure S23** The secretion of pro-inflammatory cytokines of IL-6, and IL-1β by DC2.4 cells in different treatment groups. The values of IL-6, and IL-1β concentration represent the mean of three independent experiments, and the error bars indicate the SD from the mean. Four treatment groups were employed including PBS (I), PdIr (II), OMVs (III), and PdIr@OMVs (IV). * indicates the contrasts between experimental groups and control (PBS). ^*^*P* < 0.05, ^**^*P* < 0.01, and ^***^*P* < 0.001.


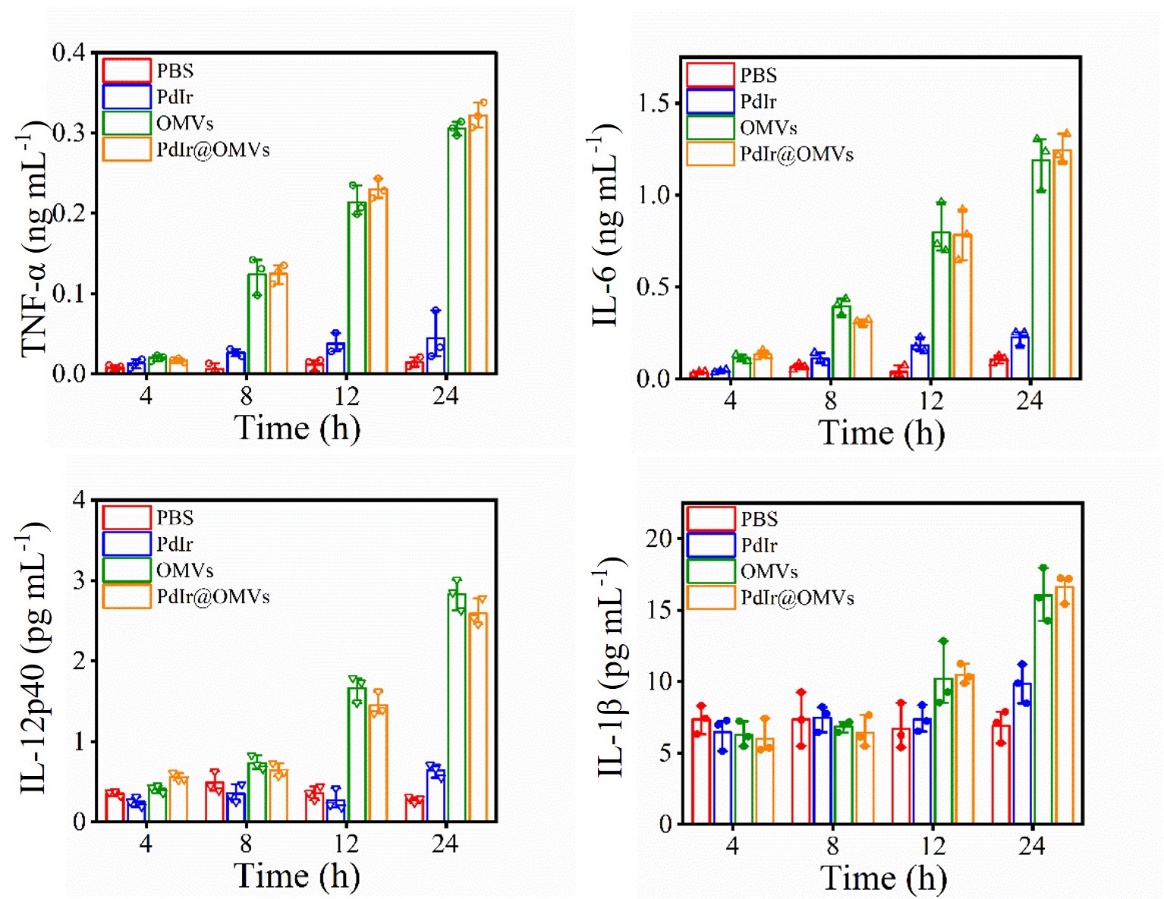


Figure S24 The secretion of pro-inflammatory cytokines of TNF-α, IL-6, IL-12p40 and IL-1β by DC2.4 cells at different time points in the four treatment groups. The values of TNF-α, IL-6, IL-12p40 and IL-1β concentration represent the mean of three independent experiments, and the error bars indicate the SD from the mean.


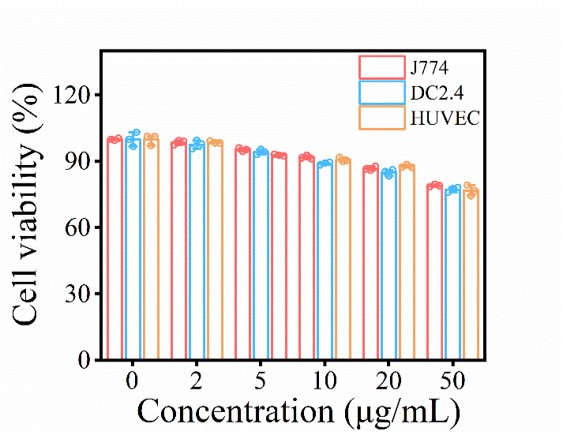


Figure S25 Viability of J774, DC2.4, and HUVEC cells after incubation with PdIr@OMVs at various concentrations for 24 h, respectively. The values of cell viability represent the mean of three independent experiments, and the error bars indicate the SD from the mean.


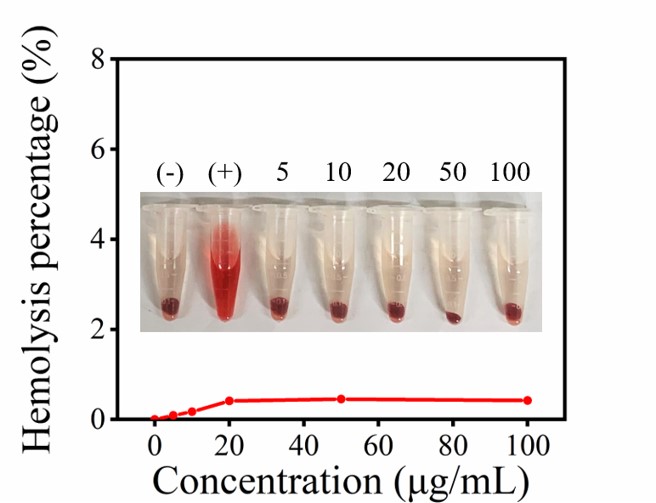


Figure S26 The haemolytic assay of red blood cells (RBC) after incubation with PdIr@OMVs at various concentrations for 4 h. In this assay, DI water and PBS buffer were used as the positive and negative control, respectively.


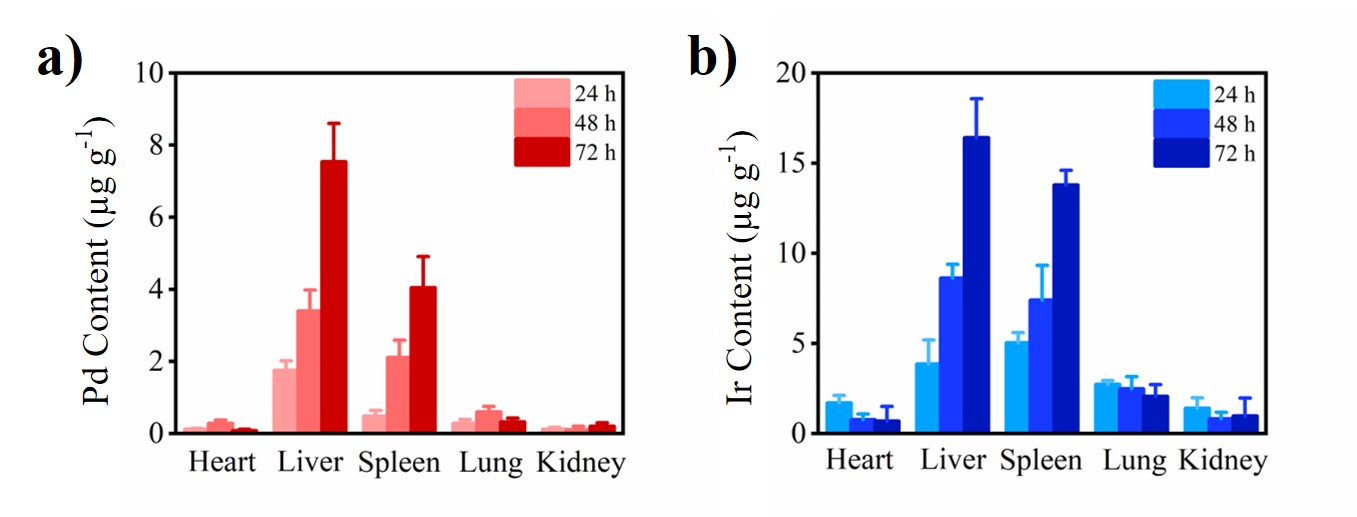


Figure S27 The distribution of Pd (a) and Ir (b) element in the organs (heart, liver, spleen, lung, and kidney) of healthy mice intravenously injected with PdIr@OMVs at the time point of 24, 48, and 72 h, respectively. The values of Pd and Ir content represent the mean of three independent experiments, and the error bars indicate the SD from the mean.


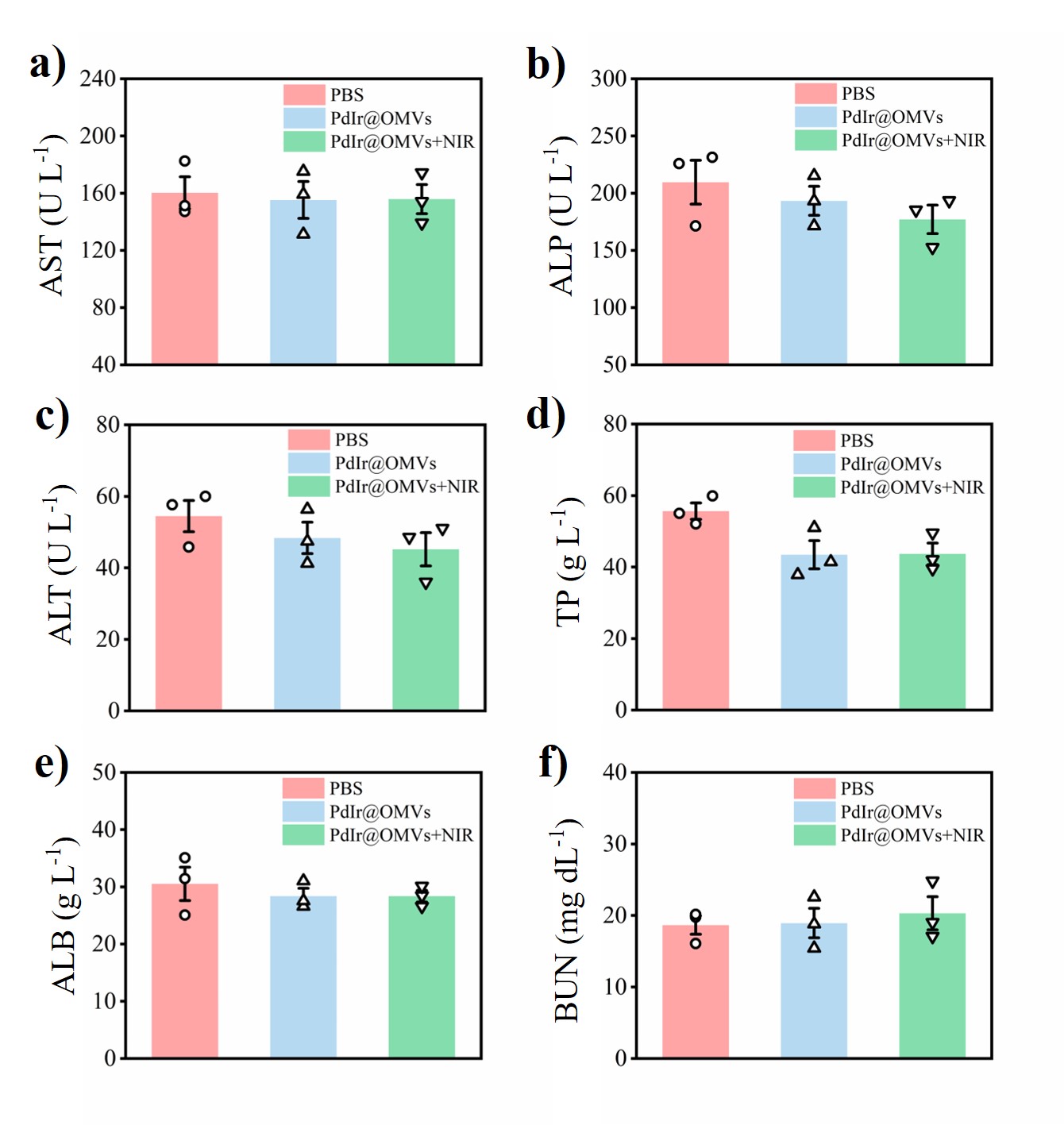


Figure S28 Blood biochemistry data of healthy mice on the 7^th^ day of postinjection of PdIr@OMVs, PdIr@OMVs + NIR, and PBS (control), respectively. In these data, AST stands for aspartate aminotransferase (a), ALP stands for alkaline phosphatase (b), ALT stands for alanine aminotransferase (c), TP stands for total protein (d), ALB stands for albumin (e), and BUN stands for blood urea nitrogen (f). The values of AST, ALP, ALT, TP, ALB, and BUN represent the mean of three independent experiments, and the error bars indicate the SD from the mean.


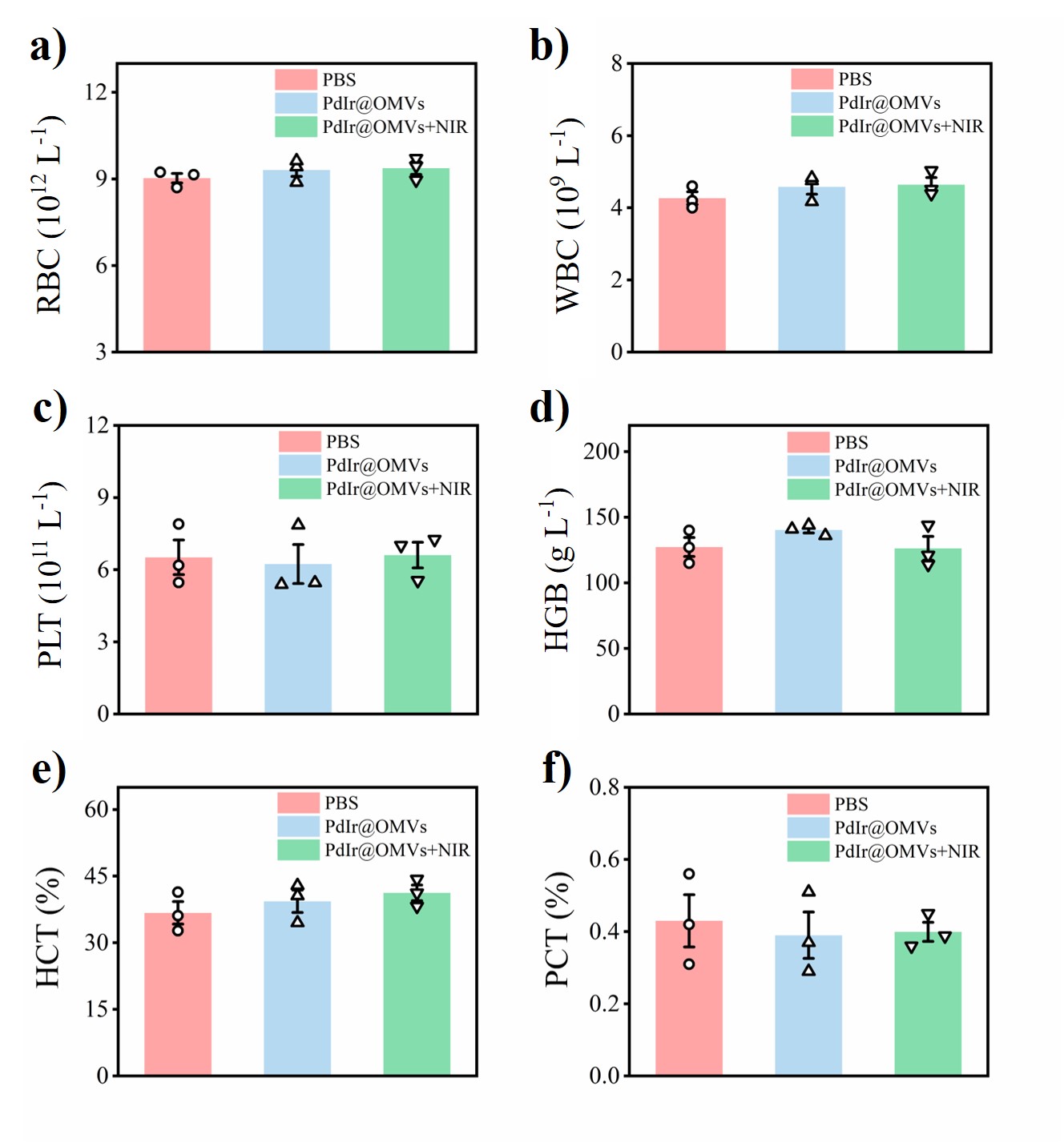


Figure S29 Blood routine data of healthy mice on the 7^th^ day of postinjection of PdIr@OMVs, PdIr@OMVs + NIR, and PBS (control), respectively. In these data, RBC stands for red blood cell (a), WBC stands for white blood cell (b), PLT stands for platelet (c), HGB stands for hemoglobin (d), HCT stands for hematocrit (e), and PCT stands for procalcitonin (f). The values of WBC, RBC, PLT, HGB, HCT, and PCT represent the mean of three independent experiments, and the error bars indicate the SD from the mean.


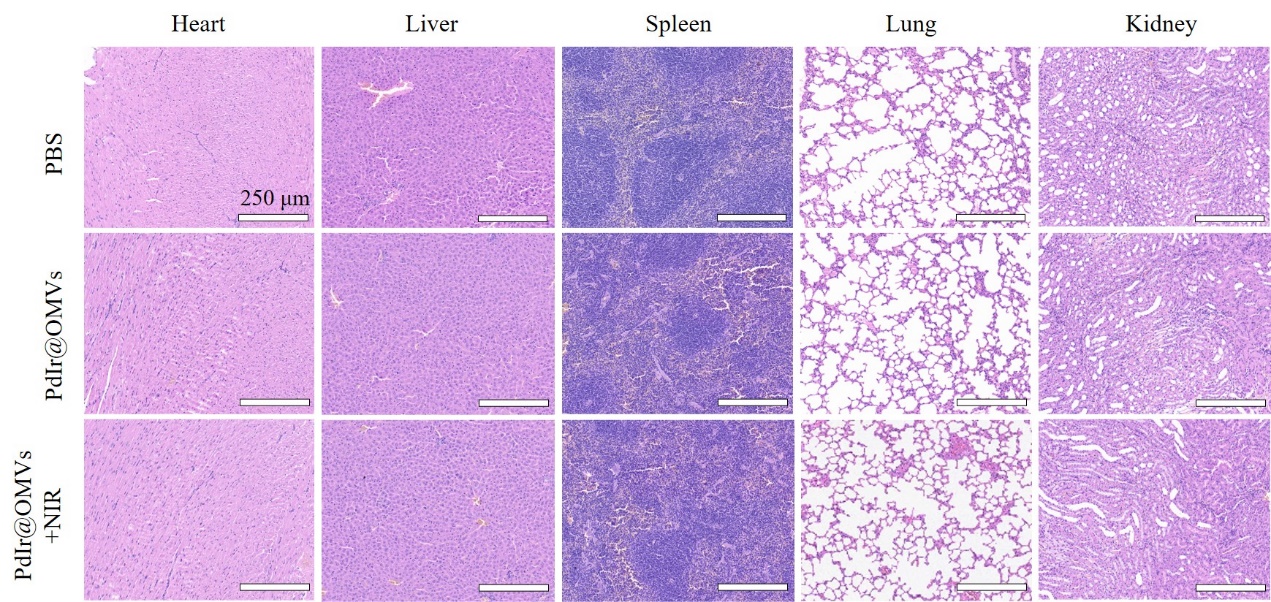


Figure S30 Representative H&E staining images of major organs (heart, liver, spleen, lung and kidney) of healthy mice on the 7^th^ day of postinjection of PdIr@OMVs, PdIr@OMVs + NIR, and PBS (control), respectively.


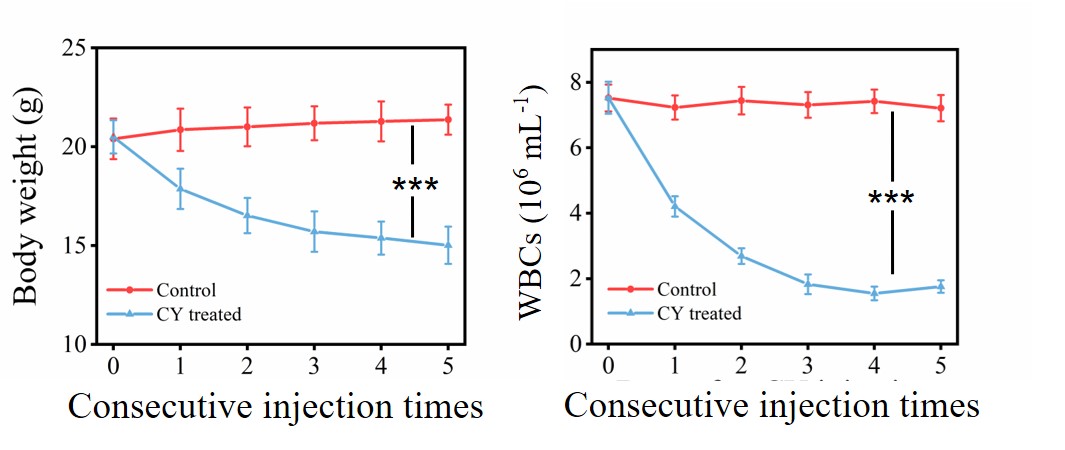


Figure S31 The curves of body weight and serum white blood cells (WBCs) number of mice with the consecutive injection times of cyclophosphamide (CY). The values of body weight and WBCs number represent the mean of three independent experiments, and the error bars indicate the SD from the mean. ^***^*P* < 0.001.


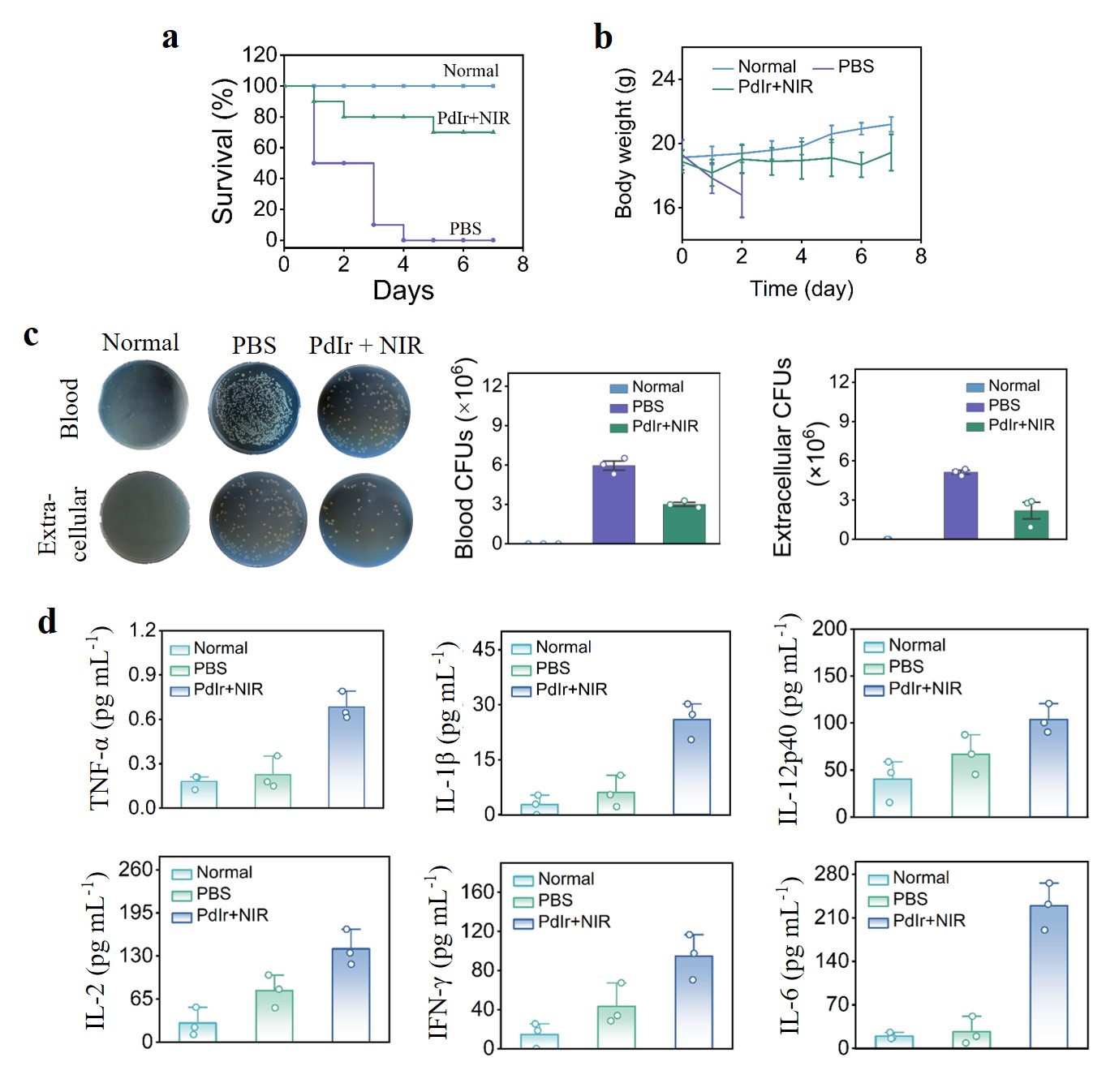


**Figure S32** (a) Survival rate and (b) body weight of immunocompromised septic mice during 7 days of different treatments. (c) Agar plate images of bacterial culture and the corresponding quantitative data of bacterial colonies extracted from the blood and peritoneal exudate of immunocompromised septic mice on the 24^th^ h of different treatments. (d) The secretion of proinflammatory cytokines retrieved from the serum of immunocompromised septic mice on the 24^th^ h of different treatments.


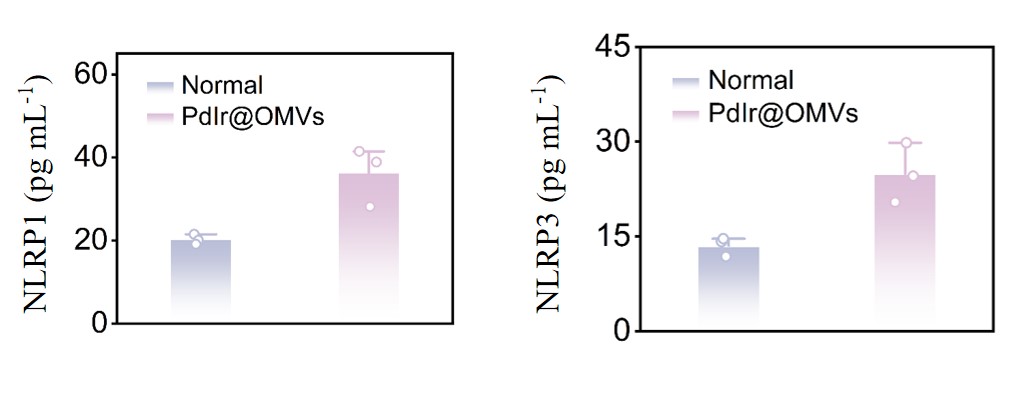


**Figure S33** The expression levels of receptors NLRP1 and NLRP3 on the surface of bone marrow neutrophils extracted from PdIr@OMVs-treated immunosuppressive septic mice.


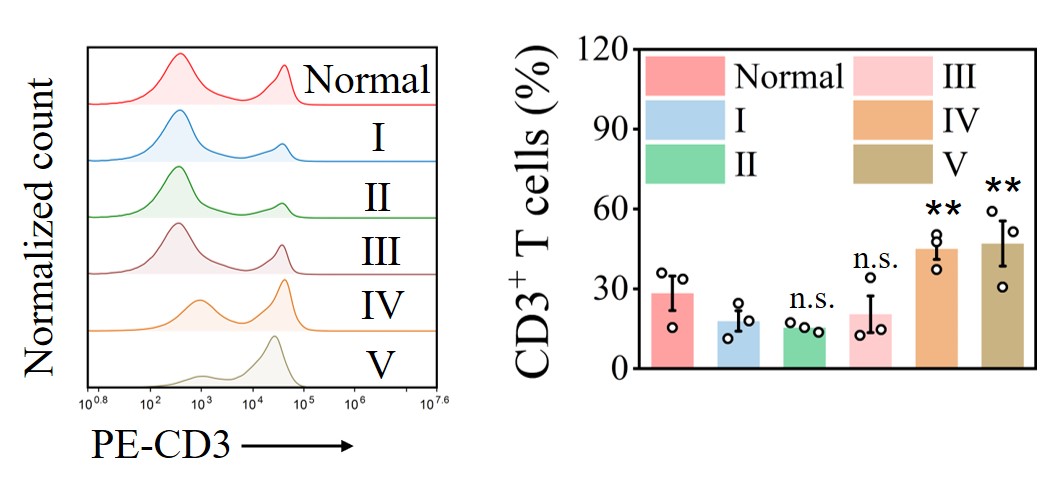


Figure S34 Representative flow cytometric plots and relative quantitative results of CD3^+^ T cells retrieved from in the spleen of immunocompromised septic mice on the 24^th^ h of different treatments. The values of CD3^+^ T cells proportion represent the mean of three independent experiments, and the error bars indicate the SD from the mean. Five treatment groups were employed including PBS (I), NIR (II), PdIr (III), PdIr@OMVs (IV), and PdIr@OMVs + NIR (V), while the group of normal healthy mice was used as the negative control. * indicates the contrasts between experimental groups and control (PBS). ^**^*P* < 0.01, and ^n.s.^*P* > 0.05.


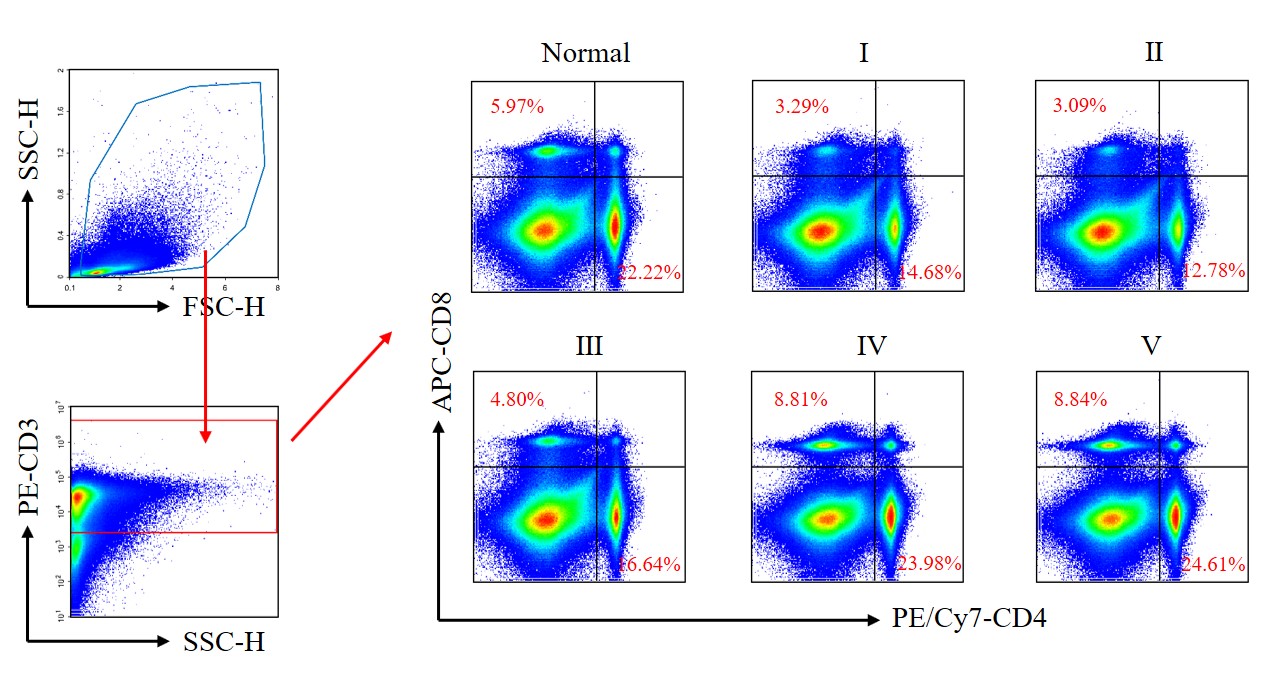


Figure S35 The gating strategy used for the flow cytometry experiments showed in Figure 7h.


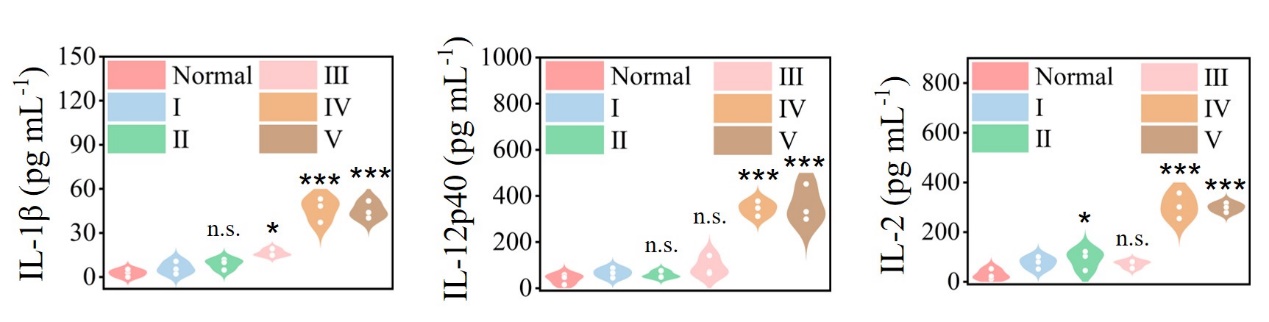


Figure S36 The secretion of pro-inflammatory cytokines of IL-1β, IL-12p40, and IL-2 retrieved from the serum of immunocompromised septic mice on the 24^th^ h of different treatments. The values of IL-1β, IL-12p40, and IL-2 concentration represent the mean of three independent experiments, and the error bars indicate the SD from the mean. Five treatment groups were employed including PBS (I), NIR (II), PdIr (III), PdIr@OMVs (IV), and PdIr@OMVs + NIR (V), while the group of normal healthy mice was used as the negative control. * indicates the contrasts between experimental groups and control (PBS). ^*^*P* < 0.05, ^***^*P* < 0.001, and ^n.s.^*P* > 0.05.


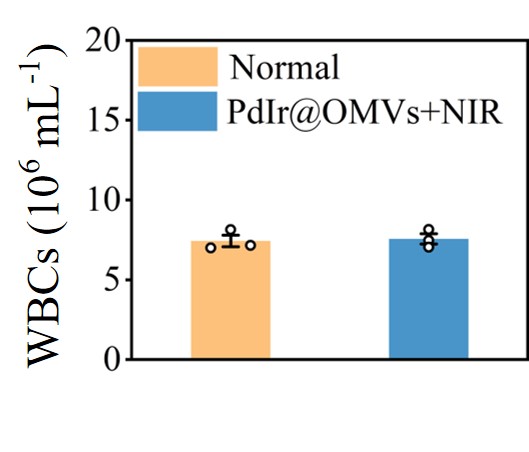


Figure S37 The WBCs number retrieved from the serum of immunocompromised septic mice on the 3^rd^ day of PdIr@OMVs + NIR treatment. The values of WBCs number represent the mean of three independent experiments, and the error bars indicate the SD from the mean. The normal healthy mice were used as the control.


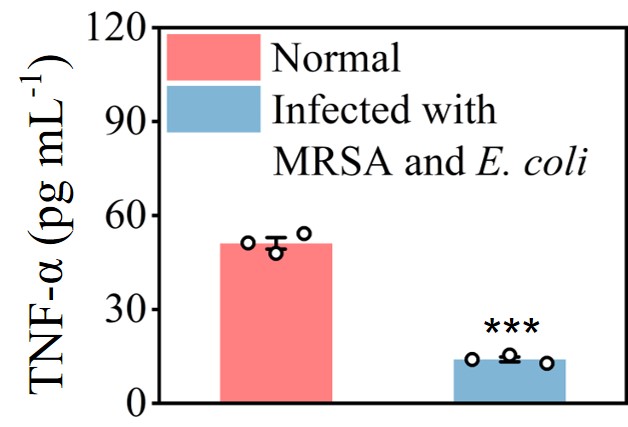


Figure S38 The secretion of pro-inflammatory cytokine of TNF-α retrieved from the serum of healthy mice on the 2^nd^ day postinjection of the mixture of MDR *E. coli* and MRSA. The values of TNF-α concentrations represent the mean of three independent experiments, and the error bars indicate the SD from the mean. The normal healthy mice were used as the control.


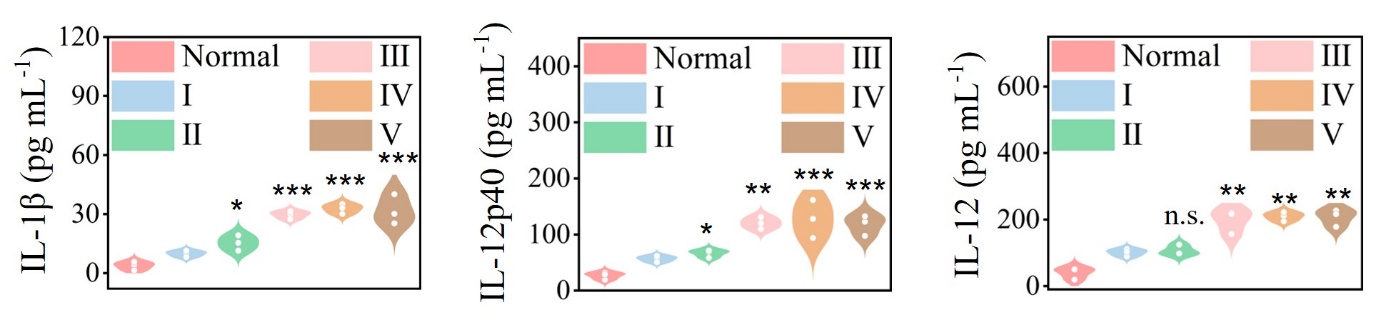


Figure S39 The secretion of pro-inflammatory cytokines of IL-1β, IL-12p40, and IL-2 retrieved from the serum of immunocompromised MDR polymicrobial septic mice on the 24^th^ h of different treatments. The values of IL-1β, IL-12p40, and IL-2 concentration represent the mean of three independent experiments, and the error bars indicate the SD from the mean. Five treatment groups were employed including PBS (I), NIR (II), PdIr (III), PdIr@OMVs (IV), and PdIr@OMVs + NIR (V), while the group of normal healthy mice was used as the negative control. * indicates the contrasts between experimental groups and control (PBS). ^*^*P* < 0.05, ^**^*P* < 0.01, ^***^*P* < 0.001, and ^n.s.^*P* > 0.05.


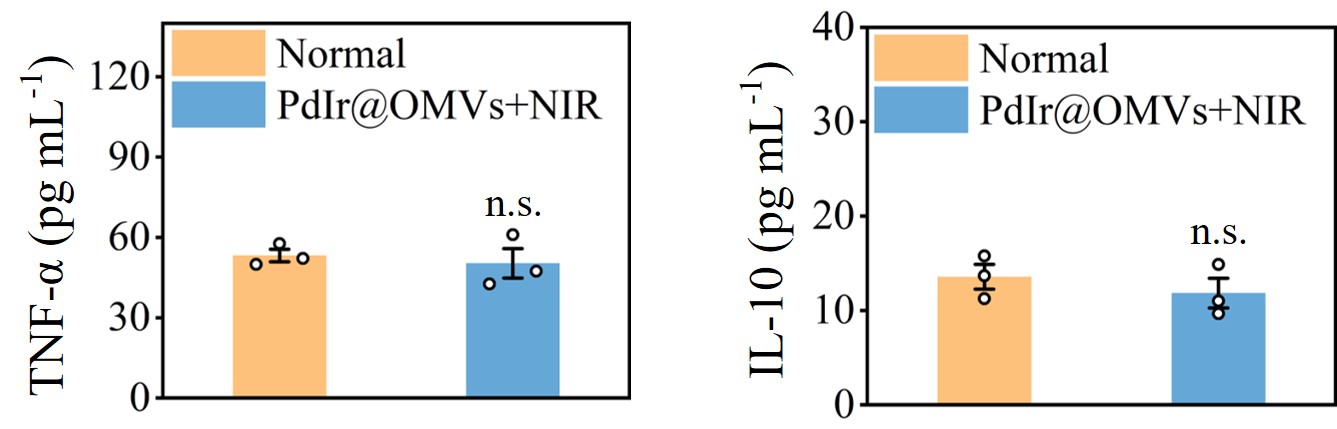


**Figure S40**  The secretion of pro-inflammatory cytokine of TNF-α and anti-inflammatory cytokine of IL-10 retrieved from the serum of immunocompromised MDR polymicrobial septic mice on the 3^rd^ day of PdIr@OMVs + NIR treatment. The values of TNF-α and IL-10 concentration represent the mean of three independent experiments, and the error bars indicate the SD from the mean. The normal healthy mice were used as the control. ^n.s.^*P* > 0.05.

Table S1. Michaelis–Menten kinetics of POD-like reaction of PdIr@OMVs.


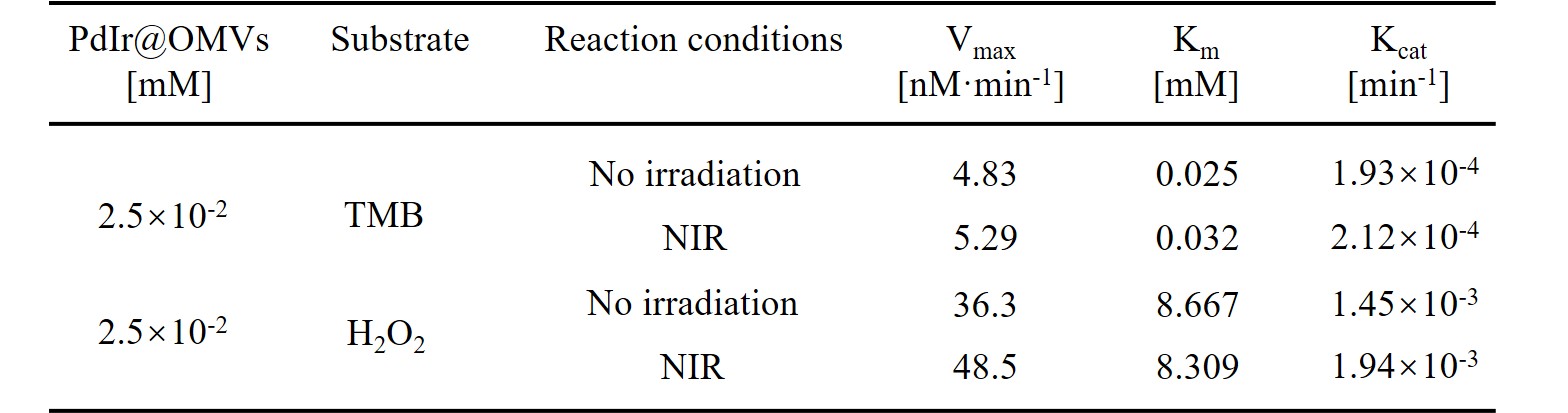

Supplement: Supplementary file 1 — exp270056‐sup‐0001‐SupMat.docx [file EXP2-5-20250127-s001.docx]
